# Versatile Membrane Deformation Potential of Activated Pacsin

**DOI:** 10.1371/journal.pone.0051628

**Published:** 2012-12-07

**Authors:** Shih Lin Goh, Qi Wang, Laura J. Byrnes, Holger Sondermann

**Affiliations:** Department of Molecular Medicine, Cornell University, Ithaca, New York, United States of America; Institut Curie, France

## Abstract

Endocytosis is a fundamental process in signaling and membrane trafficking. The formation of vesicles at the plasma membrane is mediated by the G protein dynamin that catalyzes the final fission step, the actin cytoskeleton, and proteins that sense or induce membrane curvature. One such protein, the F-BAR domain-containing protein pacsin, contributes to this process and has been shown to induce a spectrum of membrane morphologies, including tubules and tube constrictions *in vitro*. Full-length pacsin isoform 1 (pacsin-1) has reduced activity compared to its isolated F-BAR domain, implicating an inhibitory role for its C-terminal Src homology 3 (SH3) domain. Here we show that the autoinhibitory, intramolecular interactions in pacsin-1 can be released upon binding to the entire proline-rich domain (PRD) of dynamin-1, resulting in potent membrane deformation activity that is distinct from the isolated F-BAR domain. Most strikingly, we observe the generation of small, homogenous vesicles with the activated protein complex under certain experimental conditions. In addition, liposomes prepared with different methods yield distinct membrane deformation morphologies of BAR domain proteins and apparent activation barriers to pacsin-1's activity. Theoretical free energy calculations suggest bimodality of the protein-membrane system as a possible source for the different outcomes, which could account for the coexistence of energetically equivalent membrane structures induced by BAR domain-containing proteins *in vitro*. Taken together, our results suggest a versatile role for pacsin-1 in sculpting cellular membranes that is likely dependent both on protein structure and membrane properties.

## Introduction

Local differences and dynamic changes in curvature are hallmarks of cellular membranes, contributing to the identity of organelles and to mechanisms in membrane trafficking and signaling [1]. Peripheral and integral membrane proteins have been identified that either promote or stabilize membrane curvature at different locations in the cell. For example, endocytosis relies on the coordinated interplay of coat and adaptor proteins to initiate the formation and stabilization of a bud-neck structure, followed by the recruitment of the large G protein dynamin and subsequent fission [2]–[6]. In addition, reorganization of the actin cytoskeleton via the recruitment and activation of Wiskott-Alrich Syndrome proteins (WASP) provides another driving force in this process [7], [8].

Proteins containing a Bin/Amphyphysin/Rvs (BAR) domain have emerged as facilitators of membrane trafficking and fission by directly stabilizing tubular membrane structures *in vitro* and in cells [9]–[11]. They can be divided into three distinct structural classes based on their deformation activity and structures: BAR and N-BAR domain-containing proteins (e.g. endophilin, amphiphysin, sorting nexin 9, and APPL1) prefer highly curved membranes; F-BAR domain-containing proteins (e.g. CIP4, FCHo2, pacsin/syndapin) are often associated with wider tubules; and inverse or I-BAR domain-containing proteins (e.g. IRSp53; MIM) induce membrane invaginations [10], [12]–[16]. The BAR domain fold consists of three helices that form a six-helix bundle in a dimeric assembly, the predominant quaternary structure in solution [13]. The preference for distinct membrane curvatures is partially encoded in the particular folds of the different subfamilies. The dimeric BAR domains resemble an overall crescent shape. N-BAR and F-BAR domains have a concave surface lined with positively charged residues and other motifs involved in membrane interactions [13], [17]–[20]. The intrinsic curvature of N-BAR domains is higher than that of F-BAR domains characterized to date, and the lower degree of curvature of the latter often matches their preference for wider membrane tubules [21]–[23]. In contrast, the convex surface in I-BAR proteins mediates membrane interactions and promotes filopodia formation [24]. The most recent member of the I-BAR family, Pinkbar, is a unique case of a rather flat dimer that prefers flat membrane supports [25].

**Figure 1:**
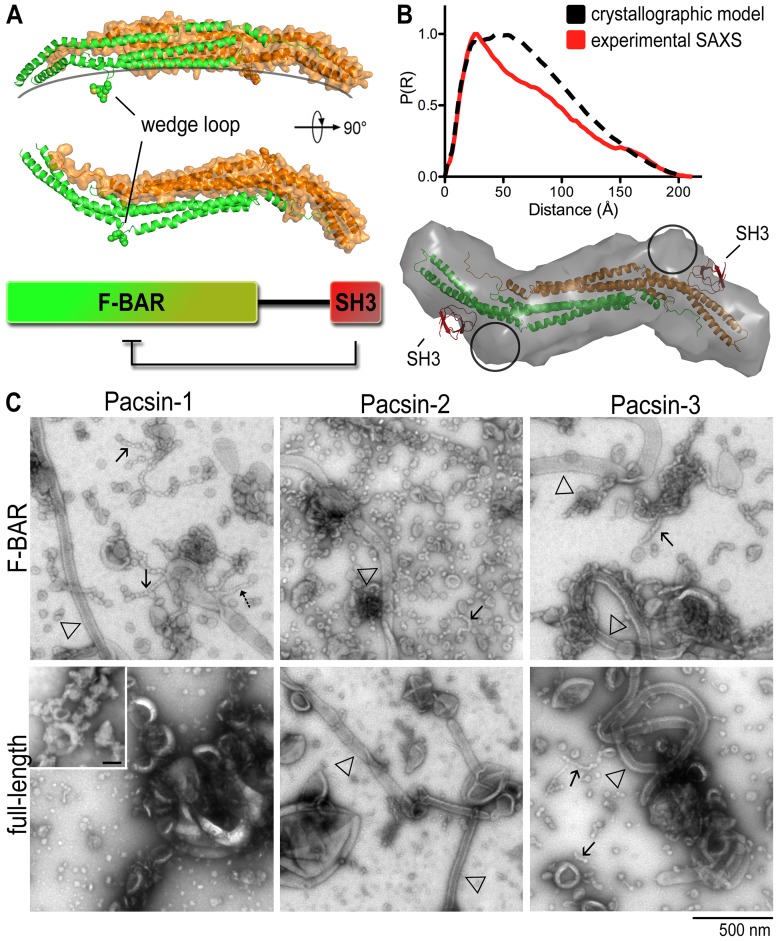
Membrane deformation by human pacsin isoforms. A. Domain organization and structure of pacsin-1. The structure shows a F-BAR domain dimer with the protomers shown in green and orange, respectively. B. SAXS-based comparison of full-length pacsin-1 in solution and in crystals. Distant distribution functions, Rg and Dmax values were determined based on the full-length crystal structure [28] and the solution scattering data [27]. R_g_/D_max_ (crystal) = 212/60 Å; R_g_/D_max_ (SAXS) = 215/58 Å. Discrepancies between the respective distance distribution functions can be explained by the flexible linkers that connect the F-BAR and SH3 domains and were not modeled in the crystal structures. C. Negative-stain electron micrographs. The membrane deformation potential of human pacsin isoforms and their isolated F-BAR domains was monitored by EM. Folch fraction I liposomes were incubated with purified proteins (5–10 µM), and processed as described in Materials and Methods. Arrows indicate specific membrane morphologies (solid arrows, pearling structures; dashed arrows, narrow tubules; open triangles, wide tubules). Inset shows liposome-only control; scale bar, 100 nm.

Recently, some exceptions to these correlations have been reported for F-BAR domain-containing proteins. In addition to its canonical function of stabilizing wide tubules, the F-BAR domain of FCHo2 also facilitates the formation of tubules with high curvature [2], [22]. srGAP2, a protein involved in neuronal migration and morphogenesis, contains an F-BAR domain based on its primary sequence, yet induces I-BAR-like membrane protrusions [26]. Another example is pacsin, also known as syndapin, which has been shown to induce a wide range of membrane deformations, including membrane tubules of various diameters, pearling structures and invaginations [27]–[29]. Structural and functional analyses revealed multiple features that may contribute to pacsin's unique morphogenetic potential [27]–[31], especially the finding that its F-BAR domain adopts a distinct lateral curvature in addition to its concave surface (Fig. 1A). These geometric constraints may contribute to the variability in pacsin-induced membrane morphologies and its potential to form different types of higher-order lattices on lipid bilayers [27]. Another striking feature is a short loop within helix 2 that forms an amphipathic wedge, proposed to dip into the acyl chain layer of one bilayer leaflet (Fig. 1A) [27]–[31]. Indeed, insertion of amphipathic helices or loops has been identified as one of the main forces in the generation of membrane curvature [17], [32], [33]. Other factors that may contribute to this activity include protein oligomerization and electrostatic interactions of the curved protein scaffold with the membrane [21], [23], [27], [34], [35].

Three isoforms of pacsin are found in mammals, with expression levels being tissue-specific: pacsin-1 is enriched in neurons, pacsin-2 is ubiquitously expressed, and pacsin-3 is found mainly in muscle [36]. All three isoforms contain a conserved C-terminal SH3 domain that interacts with the proline-rich domain (PRD) of several proteins, including those of dynamin and WASP, providing a link between endocytosis and the cytoskeleton [8], [36]–[39]. SH3 domain-mediated activation of neural WASP (N-WASP) is required for regulating actin polymerization, which is essential for proper neuromorphogenesis and cellular motility [30], [39]. On a cellular level, pacsin contributes to clathrin-dependent endocytosis and the recycling of synaptic vesicles via its SH3 domain engagement with dynamin's PRD. The pacsin-dynamin interaction is especially important during high neuronal activity, where the complex has been implicated in clathrin-independent pathways of synaptic vesicle retrieval [40]–[42]. In addition to the SH3 domain, a recent study revealed two phosphorylation sites within the F-BAR domain of pacsin-1 that can regulate its membrane sculpting potential, providing another means of pacsin-1 regulation in cells [43].

The PRD is a non-catalytic domain of dynamin located at the C-terminus of the G protein. In addition to interacting with SH3 domain-containing proteins, which targets dynamin to endocytotic sites [44]–[46], the PRD is also important for the self-assembly and self-activation of dynamin [47]. Being the most divergent region among the three dynamin isoforms, the PRD confers isoform-specific functions. Recent studies have shown that the PRD is responsible for differential self-assembly propensities and coated pit localization in an isoform-specific manner, providing tissue-specific regulation [48]–[51]. On a molecular level, the PRD binds to SH3 domain proteins via core PxxP motifs that are usually flanked by basic residues on one or both sides [46], [52].

Recently, we reported that pacsin-1 is autoinhibited *in vitro* [27], which is consistent with the suppressed activity of its fruit fly paralog *in vivo* [38]. Both studies identified the SH3 domain as an autoinhibitory feature. Based on small-angle X-ray scattering (SAXS) experiments, we proposed a compact structure for pacsin-1, in which the SH3 domains fold back onto the F-BAR dimer (Fig. 1B) [27]. Confirmation of such a model came from the crystal structure of full-length pacsin-1, validated by mutagenesis and peptide competition studies that revealed increased tubulation activity upon dislodging pacsin's SH3 domain from the F-BAR domain dimer [28].

Here, we report pacsin-1's membrane deformation ability in the presence of the full-length PRD of dynamin-1 *in vitro*, and demonstrate a role for the polybasic PRD in modulating the sculpting potential of pacsin-1. While we observed membrane tubules under certain experimental conditions, consistent with previous results, we also noted vesicle formation as a dominant feature using standard liposome preparation methods. A similar observation was made with full-length endophilin bound to the entire PRD, suggesting a more general mechanism by which membrane scaffolding and insertion mechanism could directly facilitate fission. We also reveal that membrane properties of the liposomes play an influential role in the curvature generating activities of pacsin-1. Such a notion is supported by pacsin-1's variable membrane deformation potential with liposomes prepared following different protocols, which further highlights bimodality in the protein-membrane system, and pacsin-1's potential versatility in generating or reacting to membrane curvature during membrane trafficking.

## Results

### Pacsin isoforms have different levels of membrane sculpting activity

Previously, we and others reported an SH3-dependent autoinhibition mechanism for the brain-specific pacsin-1 [27], [28], [38]. We have now extended the analysis to the other two human isoforms, pacsin-2 and 3. The purified, isolated F-BAR domains or full-length proteins were incubated with liposomes made from Folch (I) brain lipids following a standard sonication/freeze-thaw protocol, followed by visualization via negative-stain transmission electron microscopy (EM). As previously observed, the F-BAR domain of pacsin-1 (pacsin-1^F-BAR^) produced three distinct membrane morphologies under these conditions (Fig. 1C): wide tubules (open triangle), narrow tubules (dashed arrow) and pearling or beads-on-a-string structures (solid arrows). Similar narrow tubules, but with slightly larger diameter, have been noted in reactions with another F-BAR domain protein FCHo2, and have been attributed to residues predicted to mediate membrane interactions not lining up perfectly with the concave surface of the F-BAR domain [2], [22]. Such a mismatch is also the case for pacsin's F-BAR domain, and the pearling structures may represent an extreme case also driven by insertion of the wedge loop. In contrast, full-length pacsin-1 showed markedly reduced membrane deformation activity (Fig. 1C) [27].

A reduction of the membrane deformation potential of pacsin-1 can be achieved when the isolated SH3 domain was added to the F-BAR domain in trans (unpublished data), supporting the proposed model in which the SH3 domain binds to the F-BAR domain and reduces its activity [27], [28]. In fact, the molecular dimensions (R_g_, D_max_) computed based on a full-length pacsin-1 crystal structure [28] closely resemble those based on small-angle X-ray scattering [27], demonstrating that the compact, autoinhibited conformation of pacsin-1 observed in the crystalline state also dominates in solution (Fig. 1B). While the SAXS-based, low-resolution structural model of autoinhibited pacsin-1 was consistent with a compact conformation seen in the crystal structure, the SAXS-based model suggests an alternative binding site of the SH3 domain on the F-BAR domain dimer (circles in Fig. 1B) [27]. This observation could indicate multiple docking positions for the SH3 domain on the F-BAR domain, consistent with the variability observed in the crystal structures [28].

While the F-BAR domain of pacsin-2 and 3 produced similar membrane morphologies under these conditions, the autoinhibition of the full-length proteins appeared to be different from that of pacsin-1 (Fig. 1C). Full-length pacsin-2 was still able to generate wide tubules, but its vesiculation ability was observed to be impaired. On the other hand, the autoinhibition of full-length pacsin-3 appeared to be less pronounced or absent, even. A minor but noticeable difference in these micrographs is the absence of the narrow tubules that were observed with the F-BAR domain of pacsin-1 and FCHo2. A potential explanation may involve variations in the degree of lateral curvature and/or flexibility of the distal tips within the F-BAR domain of pacsin-1 and 2, as suggested by a recent crystallographic analysis of different pacsin isoforms [31]. Considering the high sequence divergence in the linker regions and the high degree of conservation of the F-BAR and SH3 domains, the nature of the linker segment may also contribute to the different morphogenic potential and degree of autoregulation among the isoforms.

### Activation of full-length pacsin-1 by dynamin-1 PRD

**Figure 2:**
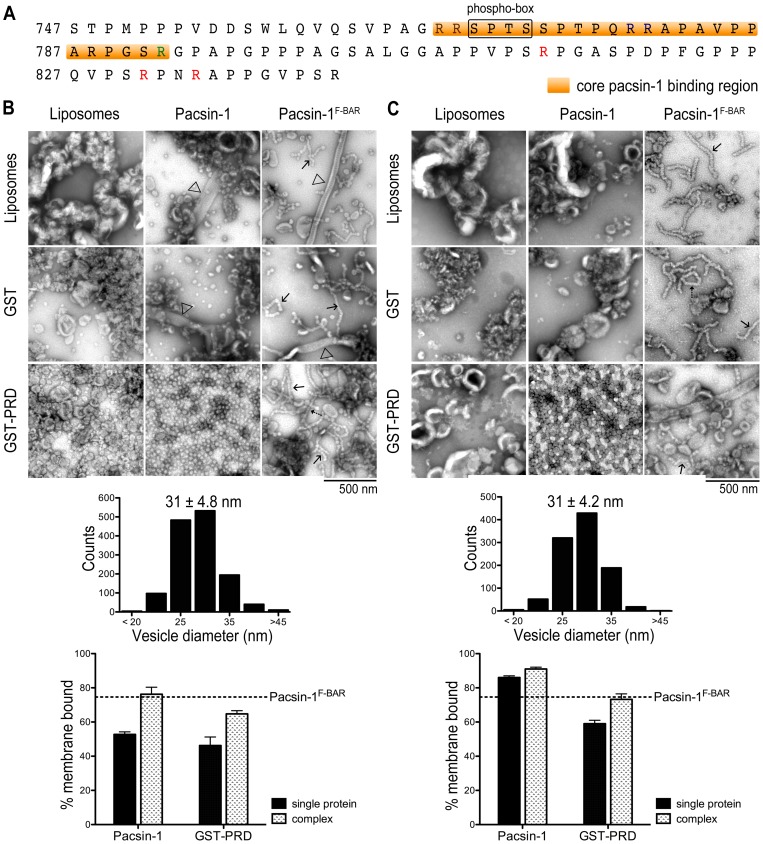
Activation of pacsin-1 by the proline-rich domain (PRD) of dynamin-1. A. Sequence of the mouse dynamin-1 PRD. A regulatory sequence (phospho-box), the core pacsin-1 binding region (orange) and arginine residues are highlighted. The sequence is 100% identical to the human dynamin-1 PRD. The mouse PRD was expressed as GST-fusion protein. B. Negative-stain EM with Folch liposomes. Liposomes were imaged as described before following incubations with the indicated proteins and protein complexes (top panel). The histogram (middle panel) shows the size distribution of the vesicles produced by pacsin-1 in the presence of GST-PRD. Vesicle diameters were quantified from electron micrographs taken from three independent experiments. Liposome-protein co-pelleting assays (bottom panel) were used to assess the amount of protein bound to lipid vesicles. The horizontal, dashed line indicates the lipid-bound fraction of the isolated pacsin-1 F-BAR domain under similar conditions. Two-tailed unpaired t-tests for both pacsin-1 and GST-PRD were p<0.05, N = 4. C. Negative-stain EM with synthetic lipid mixtures. Experiments were carried out as described in (B), but using liposomes with the composition POPC/ POPE/ POPS = 27.5/27.5/45. Error bars represent standard deviations of a minimum of 3 independent experiments.

The SH3 domain of pacsin binds to the PRD of proteins involved in endocytosis such as dynamin, synaptojanin and WASP/N-WASP [53], [54]. Incubation of pacsin-1 with a minimal PRD peptide from dynamin increases its tubulation activity *in vitro* [28], indicating that PRD-SH3 domain interactions relieve the intra-molecular, autoinhibited conformation in full-length pacsin-1, likely by releasing the SH3 domains from the F-BAR dimer. The previous studies were conducted with a minimal peptide of the PRD that has reduced affinity for pacsin compared to the entire PRD [28]. Here, we used the full-length PRD of dynamin-1 that includes the full binding sequence (Fig. 2A) [52]. In addition, liposomes were prepared either with Folch (I) lipids or with a synthetic lipid mixture that have properties resembling the lipid composition in the inner leaflet of the plasma membrane [55], [56]. Liposomes were incubated with full-length pacsin-1 in the presence or absence of dynamin-1 PRD fused to GST (GST-PRD). As a control, the isolated GST moiety was co-incubated with pacsin-1 and liposomes.

**Figure 3:**
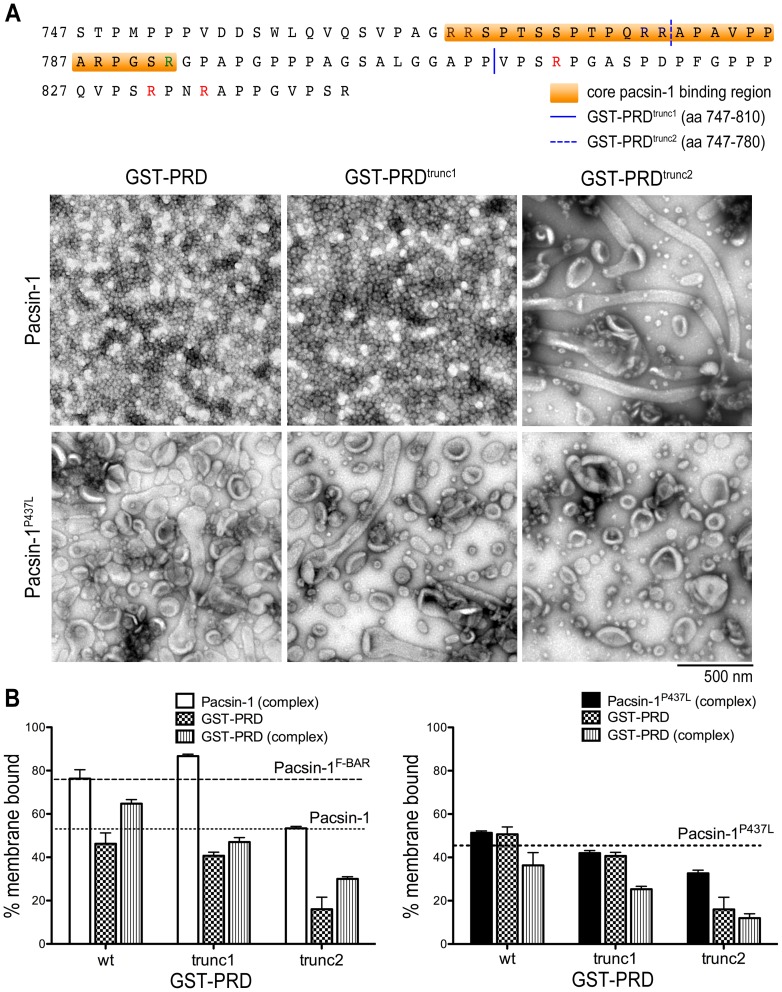
Effect of GST-PRD truncation mutants on the membrane deformation activity of pacsin-1. A. Membrane deformation of Folch liposomes. The sequences of mouse dynamin-1 PRD truncation mutants GST-PRD^trunc1^ and GST-PRD^trunc2^ are shown (top panel). Negative-stain EM images are shown after incubation of liposomes with the indicated protein complexes. Either wild-type human pacsin-1 or a corresponding protein with a single-point mutation in the SH3 domain (pacsin-1^P437L^) was used. B. Liposome co-pelleting assay. Liposome binding assays were carried out with the complexes used in (A). The horizontal, dashed lines indicate the lipid-bound fraction of the isolated pacsin-1 F-BAR domain and isolated full-length pacsin-1 under similar conditions. Error bars represent standard deviations of a minimum of 3 independent experiments.

Full-length pacsin-1 maintained minimal tubulation activity in the presence of GST, similar to findings with pacsin-1 alone (Fig. 2B). Also, the isolated F-BAR domain was insensitive to the presence of GST or GST-PRD. In stark contrast, addition of GST-PRD to full-length pacsin-1 resulted in the appearance of vesicular structures in the micrographs. The morphology of the vesicles was homogeneous, with an average diameter of 31±4.8 nm (Fig. 2B), and distinct from the tubules reported for mouse pacsin-1-PRD peptide complexes in cells or with phosphatidylserine liposomes [28]. The abundance of vesicles is dependent on relative pacsin-1 and GST-PRD concentrations in the reaction, with no tubular structures being observed at any protein concentration under these conditions (Fig. S1). Similar results were obtained when a SUMO moiety was used as the fusion partner for the dynamin-1 PRD (Fig. S2A). While GST has the propensity to form dimers, the SUMO moiety is believed to present the PRD as a monomeric ligand, indicating that a simple binding mode between the SH3 domain and the PRD is responsible for modulating pacsin-1's membrane deformation activity. Furthermore, GST-PRD and pacsin-1 co-migrate in size-exclusion chromatography (SEC), indicating the formation of a stable complex (Fig. S3A). Only a minor fraction, as indicated by the shoulder preceding the main peak appears to form higher-order complexes, most likely corresponding to a complex formed between the PRD and pacsin-1 in a tetrameric form. On the other hand, mutation of the central proline residue in the ligand-binding site of pacsin's SH3 domain (pacsin-1^P437L^) [53] prevented PRD binding, resulting in absence of stable complex formation (Fig. S3B). Consequently, small homogeneous vesicles were not observed in liposome incubations in the presence of both pacsin-1^P437L^ and GST-PRD (Fig. 3A), confirming the importance of direct SH3-PRD interactions in stimulating pacsin-1's vesiculation activity. In addition, we also observed that mutation of a critical methionine residue on the pacsin-1 wedge loop to lysine (pacsin-1^M126K^, [27]) suppressed vesiculation activity in the presence of GST-PRD (Fig. S4), corroborating our previous conclusions about the role of the wedge loop in pacsin's membrane sculpting potential [27].

Although Folch (I) lipids represent a natural and widely used lipid mixture, its uncertainty in composition and potential batch-to-batch variability pose concerns. We repeated the membrane deformation assays described above with a synthetic lipid mixture (POPC/POPE/POPS  =  27.5/27.5/45). We found that the lipid deformation activities of pacsin-1 and pacsin-1^F-BAR^ were rather similar to those observed with Folch (I) liposomes. Pacsin-1^F-BAR^ almost exclusively produced pearling structures, which at times resembled less sharply defined narrow tubules, whereas full-length pacsin-1 remained relatively inactive (Fig. 2C). The addition of GST-PRD resulted in the appearance of homogenous vesicles with a mean diameter of 31±4.2 nm (Fig. 2C). Student's t-test revealed that they were no different from the ones generated in Folch (I) lipids (p = 0.89, two-tailed unpaired, 1000<N<1400). Analogous to experiments conducted in Folch lipids, no vesiculation was observed when GST-PRD was added to the SH3-binding mutant pacsin-1^P437L^ (unpublished data).

Previous studies converged on a model by which the binding of the PRD to pacsin-1 sequesters the SH3 domains from the F-BAR domains, leading to an increased membrane sculpting potential of pacsin-1 [27], [28]. To investigate whether this activation step also resulted in increased membrane affinity of pacsin-1, we performed liposome co-pelleting assays using Folch (I) liposomes. The fractions (%) of proteins found in the pellet (membrane bound) and in the supernatant were detected using SYPRO Ruby gel stain and quantified in ImageJ (see Fig. S5A for representative of gels). Our analyses revealed that only 53% of full-length pacsin-1 was membrane bound, compared to 75% of the isolated F-BAR (Fig. 2B). GST-PRD alone also possesses appreciable membrane binding affinity, with 46% found in the membrane fraction. When GST-PRD is co-incubated with full-length pacsin-1 and liposomes, the membrane-bound fractions of both pacsin-1 and GST-PRD increased significantly to 76% and 65%, respectively (Fig. 2B; student's t-test, p<0.05). On the other hand, the membrane bound fraction of pacsin-1^P437L^ was 46%, and remained relatively unchanged (51%) when GST-PRD is added (Fig. 3B). Similarly, the presence of GST-PRD had no effect on the membrane affinity of pacsin-1^F-BAR^ (unpublished data). While this assay may not distinguish between enhanced direct protein-membrane interactions and increased protein tethering associations at the membrane (between pacsin-1 and GST-PRD), it still represented enhanced recruitment of both proteins only in the presence of each other. These results were recapitulated in liposome flotation assays using a sucrose gradient (Fig. S6A–B), further corroborating the increased membrane affinities of full-length pacsin-1 and GST-PRD upon co-incubation with Folch liposomes. A comparable trend was also observed using synthetic lipid mixtures (Fig. 2C).

### Basic residues within the dynamin-1 PRD are required for full activation of pacsin-1

Activation of pacsin-1 upon addition of a shorter dynamin-1-derived PRD peptide (residue 769–790) has been previously reported, where the activated pacsin-1 generated tubules *in vitro* [28]. Under the conditions used here, where the entire PRD of dynamin-1 was employed, vesicles appeared to be the dominant morphology observed in electron micrographs. This apparent discrepancy of membrane morphologies could originate from the differences in the PRD construct used and/or in experimental conditions, such as protein concentrations, liposome compositions and liposome physical properties. The PRD of dynamin-1 contains several PxxP motifs and has a polybasic nature, among which residues 768–792 have been shown to be important for high-affinity pacsin-dynamin interactions [52]. To determine whether specific segments of the PRD are responsible for full activation of pacsin-1, we created two truncation mutants of GST-PRD (Fig. 3A) and performed *in vitro* membrane deformation assays.

Incubation of pacsin-1 with a truncated PRD construct that removes part of the core binding segment (GST-PRD^trunc2^; aa747–780) completely abolished pacsin-1's ability to generate vesicles. Instead, only tubules were observed on the micrographs (Fig. 3A). In contrast, when GST-PRD was shortened at its C-terminus by only 30 amino acids (GST-PRD^trunc1^; aa747–810), pacsin-1 still maintained its ability to generate homogeneous vesicles that are indistinguishable from the ones generated in the presence of the entire GST-PRD (Fig. 3A, Fig. S7A). Furthermore, co-pelleting assays of Folch liposomes revealed an increase in the fraction of membrane bound pacsin-1 in the presence of GST-PRD^trunc1^, but not in the presence of the shorter PRD construct, GST-PRD^trunc2^ (Fig. 3B). Liposome flotation assays corroborated these results (Fig. S6C). Our results are consistent with earlier reports, which identified residues 768–792 in the PRD as being responsible for pacsin-1's interaction with dynamin-1, and also indicate that arginines and PxxP motifs that lie within the last 30 residues of GST-PRD were dispensable for pacsin-1's vesiculation activity.

**Figure 4:**
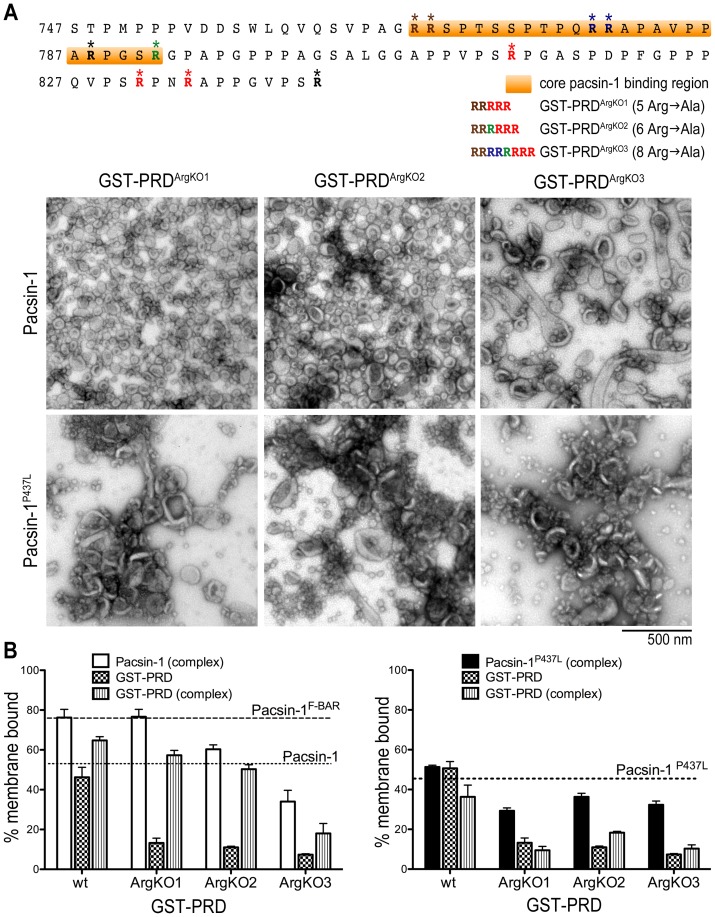
The role of arginine residues within the dynamin-1 PRD in pacsin-1 '**s membrane deformation potential.** A. Membrane deformation of Folch liposomes. The positions of Arg-to-Ala mutations (GST-PRD^ArgKO1^, GST-PRD^ArgKO2^ and GST-PRD^ArgKO3^) in the mouse dynamin-1 PRD protein sequence are shown (top panel). Negative-stain EM images are shown after incubation of liposomes with the indicated protein complexes. B. Liposome co-pelleting assay. Liposome binding assays were carried out as described in Fig. 3. Error bars represent standard deviations of a minimum of 3 independent experiments.

As an alternative strategy to determine whether the polybasic nature of the PRD is important for pacsin's vesiculation activity, we generated various GST-PRD mutants that contain arginine-to-alanine point mutations (Fig. 4A). Using full-length GST-PRD with neutralizing mutations within the last 30 residues, we first mutated the two arginines immediately upstream of the regulatory phospho-box (GST-PRD^ArgKO1^, Fig. 4A). Upon incubation with pacsin-1, we observed that pacsin-1 lost its ability to generate 31 nm-vesicles (Fig. 4A). The average vesicle size was 46±12 nm, and the overall size distribution was broader compared to experiments carried out with wild-type GST-PRD (Fig. 4A, Fig. S7B). Further mutations of arginine residues that reside within the proposed core binding sequence of GST-PRD (GST-PRD^ArgKO2^) resulted in increased heterogeneity of vesicle sizes produced by pacsin-1, whereby the mean diameters were significantly different from those induced by wild-type GST-PRD (Fig. 4A). Only occasional tubules were observed when pacsin-1 was incubated with the most neutralized mutant, GST-PRD^ArgKO3^ (Fig. 4A). In addition, pelleting assays revealed a gradual decrease in the fraction of membrane-bound pacsin-1 (and GST-PRD) as arginine residues were sequentially neutralized in the mutants GST-PRD^ArgKO1^, GST-PRD^ArgKO2^ and GST-PRD^ArgKO3^ (Fig. 4B).

**Figure 5:**
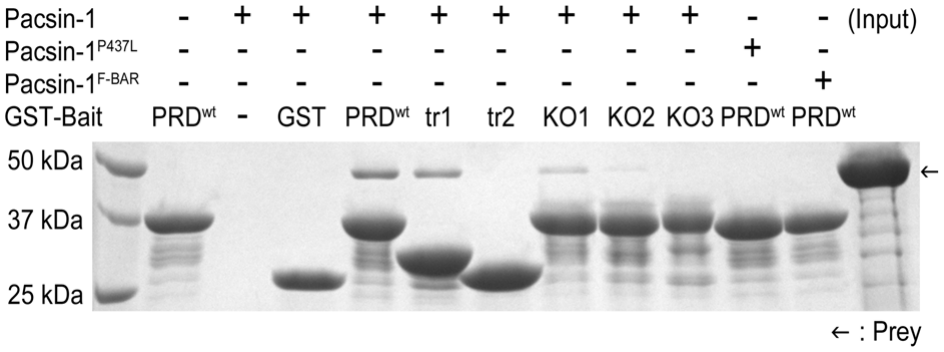
Pacsin-PRD complex formation. GST pull-down experiments were carried out by using wild-type and mutant forms of GST-PRD to examine their interactions with pacsin-1. Complexes were eluted and analyzed by SDS-PAGE and Coomassie-staining. Bait proteins: wild-type GST-PRD (PRD^wt^), GST-PRD^trunc1^ (tr1), GST-PRD^trunc2^ (tr2), GST-PRD^ArgKO1^ (KO1), GST-PRD^ArgKO2^ (KO2), GST-PRD^ArgKO3^ (KO3) and GST (negative control).

To examine the effect of various GST-PRD mutants on SH3-PRD affinity, we conducted GST pull-down experiments. For the truncation mutants, we observed weaker binding between pacsin-1 and GST-PRD^trunc1^ compared to the entire GST-PRD, and essentially no interaction between pacsin-1 and GST-PRD^trunc2^ (Fig. 5). Since the truncation in GST-PRD^trunc2^ removed some of the validated core binding sequences, the lack of strong interaction between pacsin-1 and GST-PRD^trunc2^ was expected. Pull-down data for the arginine-to-alanine point mutants corroborated pelleting assay results, whereby sequential neutralization of arginine residues in the PRD mutants resulted in their decreased affinities for pacsin-1 (Fig. 5). Altogether, we confirmed that within the entire GST-PRD, neutralizing mutations on arginine residues residing within the core binding motif (aa768–792) disrupted pacsin-PRD interactions, which correlated with the gradual loss of pacsin-1's ability to generate vesicles as the number of mutations increased. Based on our mutagenesis study, the polybasic nature of the entire PRD contributes mainly towards interaction of the PRD with pacsin-1. High-affinity binding of the SH3 of pacsin-1 to intact core binding sequences in the PRD, and the resulting higher efficiency in membrane recruitment of the complex establishes its enhanced membrane sculpting activity.

### GST-PRD also modulates membrane deformation activity of endophilin-A1

**Figure 6:**
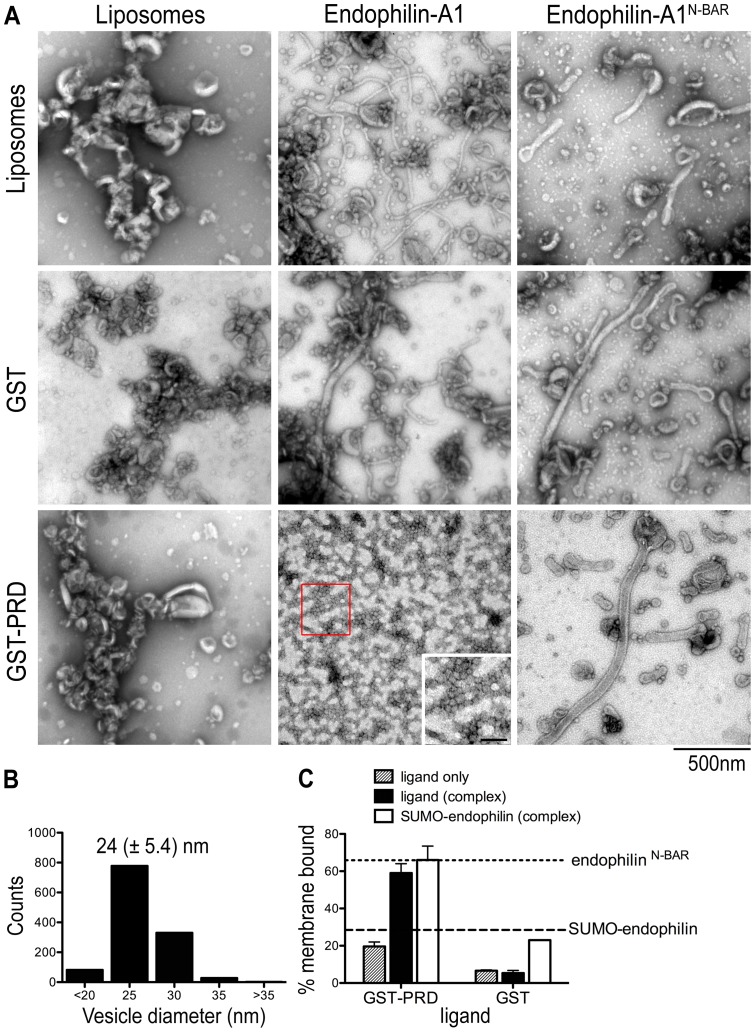
Activation of full-length endophilin-A1 by GST-PRD. A. Negative-stain EM with Folch liposomes. Assays with endophilin-A1 (full-length or N-BAR domain; 10 µM) were carried out as described before. The inset shows a zoomed-in view of the red box area of the image, with scale bar  =  100 nm. B. Statistical analysis of vesicle size distribution. Diameters of vesicles produced by endophilin in the presence of GST-PRD were quantified from electron micrographs taken from three independent experiments. C. Liposome co-pelleting assay with Folch liposomes. Liposome binding assays were carried out as described in Fig. 3. The horizontal, dashed lines indicate the lipid-bound fraction of the isolated endophilin-A1 N-BAR (expressed as His_6_-SUMO-fusion protein) domain and isolated full-length endophilin-A1 under similar conditions. Error bars represent standard deviations of a minimum of 3 independent experiments.

The PRD of dynamin has been implicated in many studies to interact with SH3 domains of various proteins, one of which is another BAR domain protein, endophilin-A1 [57]. Endophilin-A1 is enriched in neurons and functions in the recycling of synaptic vesicles [12], [17], [58], [59], where it has been shown recently to couple fission with clathrin uncoating events via its SH3 domain-mediated interactions with synaptojanin and dynamin [60]. Similar to pacsin-1, endophilin has an N-terminal membrane binding and curvature-inducing module, an N-BAR domain that is connected to the C-terminal SH3 domain via a linker peptide [58]. Consistent with previous reports [12], [17], [19], both endophilin full-length and its isolated N-BAR domain (endophilin^N-BAR^) are able to generate tubules from Folch liposomes *in vitro* (Fig. 6A). The tubules produced by full-length endophilin were on average narrower than tubules produced by endophilin^N-BAR^ (30 nm vs. 45 nm).

When endophilin-A1 was incubated with GST-PRD, we observed a switch from tubulation to vesiculation activity (Fig. 6A). The vesicles were homogeneous, with a mean diameter of 24±2.7 nm, which was smaller than vesicles generated by pacsin-1 (Fig. 6B). This could reflect the intrinsic structural differences of the two proteins, with the endophilin N-BAR domain adopting a higher degree of curvature than the F-BAR domain of pacsin. Unlike the small vesicles that were generated by endophilin^N-BAR^ at high protein concentrations [13], vesiculation here appeared not to be caused by use of excess protein since vesicles were the main morphology observed over a wide range of protein concentrations under similar experimental conditions (unpublished data). Vesiculation required the presence of the SH3 domain, as incubations of endophilin^N-BAR^ with GST-PRD did not result in vesicle generation (Fig. 6A).

The effect of endophilin-PRD complex formation on the membrane affinity of endophilin was examined using co-pelleting assays. Since full-length endophilin has a molecular weight close to GST-PRD, we expressed and purified endophilin fused to an N-terminal SUMO moiety (SUMO-endophilin) to enable analysis in pelleting assays. SUMO-endophilin still retained tubulation activity and co-migrated with GST-PRD as a stable complex in SEC experiments (unpublished data). Similar to experiments with pacsin-1, pelleting assays revealed increased membrane binding of endophilin and GST-PRD as a complex compared to their membrane affinities as separate entities (Fig. 6C). This was not observed when GST was used as the ligand (Fig. 6C). Taken together, the functional and binding assays impart similar influences of GST-PRD on the membrane sculpting potential of endophilin and pacsin-1, suggesting a more general effect of dynamin-1's PRD on its BAR-SH3 domain-containing binding partners.

### Liposome properties impact membrane deformation abilities of BAR domain proteins

Cellular membranes are subject to constant dynamic changes and alterations in response to cellular events. Heterogeneity in lipid composition and curvature could give rise to a wide range of membrane physical properties that may influence the sculpting potential of BAR/F-BAR domain proteins. Pacsin-1 has been reported to produce a large spectrum of membrane morphologies *in vitro*, ranging from vesicular structures to tubules of varying diameter [27]–[29], which could be influenced by the properties of the membrane support. Here, we asked whether the physical properties of the liposomes could account for the generation of different morphologies. Factors such as preparation methods, buffer ionic strength, multivalent cations, and lipid composition have been known to affect membrane curvature (liposome size) and lamellarity [61], [62], which could in turn affect membrane bending elasticity [32], binding affinities of proteins, and thus, membrane sculpting abilities of BAR domain proteins. Here, we concentrated on the effects of different liposome preparation methods on membrane deformation activities of BAR domain proteins.

**Figure 7:**
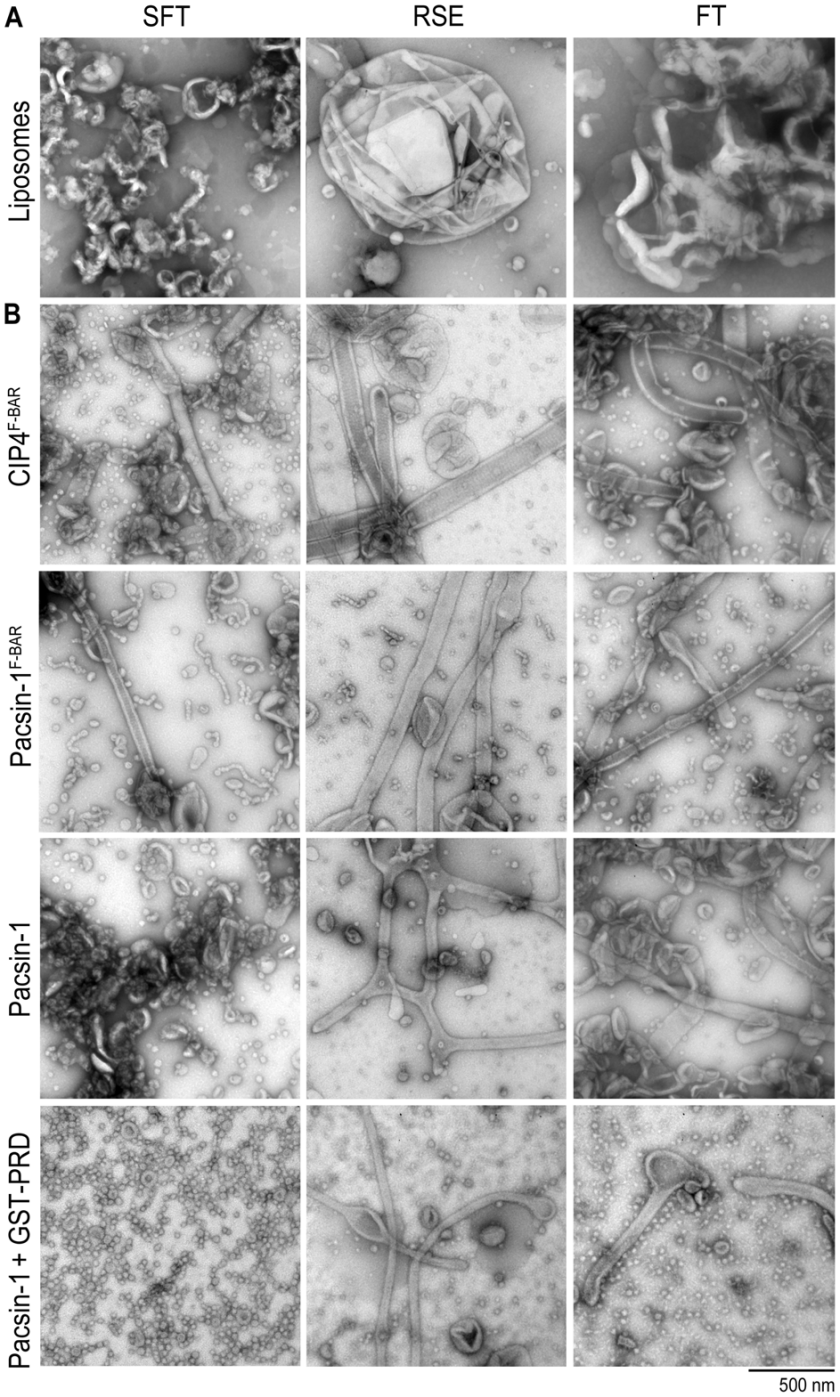
Effect of liposome preparation method on protein-induced membrane deformation. A. Negative-stain EM of liposomes prepared via sonication/ freeze-thaw (SFT), rapid solvent exchange (RSE), and freeze-thaw (FT) methods. B. Membrane deformation activities of various pacsin-1 constructs (5–10 µM) and CIP4^F-BAR^ (10 µM) in Folch liposomes prepared following three different methods. Incubations and imaging were carried out as described previously.

We employed three distinct methods for comparison. The protocol used thus far to generate liposomes involved the rehydration of a dried lipid film in aqueous buffer, followed by brief sonication and freeze-thaw cycles (“SFT method”) [23]. Sonication produces small unilamellar liposomes (with an average diameter of 20–30 nm), whereas freeze-thaw cycles equilibrate ions across membranes, which leads to fusion of bilayers to form larger liposomes [61]. The SFT method likely produces liposomes with a range of sizes and lamellarity; electron micrographs revealed that a large percentage of the liposomes were unilamellar and less than one micron in diameter, consistent with dynamic light scattering (DLS) data (Fig. 7A; unpublished data). Another method generally used to prepare hydrated bilayers from a dry film deposition involves no sonication, but only freezing and thawing of a hydrated suspension of lipids (“FT method”) [63]. This second method produces multi-lamellar liposomes of a wide range of sizes, from <100 nm to few microns, as determined by DLS (Fig. 7A, Fig. S8A). The third method, rapid solvent exchange (“RSE method”), allows a fast exchange of organic solvent with aqueous buffer, avoiding the dry film state that can cause artifactual de-mixing of lipids [64]. The size range of RSE liposomes is similar to that of liposomes prepared by the FT method (Fig. 7A, Fig. S8B), but with a lower average lamellarity of ∼1.5 [64].

*In vitro* membrane deformation experiments were performed with Folch lipids using liposomes prepared from the three methods described above, where we compared the membrane deformation activities of pacsin-1^F-BAR^, full-length pacsin-1, and pacsin-1 in the presence of GST-PRD. In addition, we included a canonical F-BAR domain, that of CIP4 (CIP4^F-BAR^), which has been reported to stabilize relatively wide membrane tubules [21]. Consistent with previous observations, CIP4^F-BAR^ more efficiently generated tubules from larger liposomes (in RSE and FT preparations) than from SFT liposomes (Fig. 7B). Pacsin-1^F-BAR^ produced both tubules and pearling structures in all three liposome preparations, but wide tubules (and shorter pearlings) dominated in RSE and FT liposomes (Fig. 7B). Unexpectedly, despite its previously reported autoinhibited membrane deformation activity, full-length pacsin-1 displayed noticeable tubulation activities in RSE and FT liposomes even in the absence of GST-PRD (Fig. 7B). The variable extent of membrane sculpting activity of pacsin-1 in the three types of liposome preparations could indicate an initial preference for shallower membrane curvature of the full-length protein, which is more abundant in RSE and FT liposome preparations.

The membrane deformation activity of pacsin-1 in the presence of GST-PRD was also examined in the three different liposome preparations. Both tubules and vesicles were observed in RSE and FT liposomes, compared to SFT liposomes that yielded only small vesicles (Fig. 7B). However, the morphology of the vesicles was different from that generated from SFT liposomes: the former have less-defined perimeters, especially those generated from RSE liposomes. RSE and FT preparations also yielded vesicles at higher pacsin-PRD concentrations (≥10 μM) whereas tubules were found at all protein concentrations (unpublished data), which may indicate a concentration-dependent phenomenon similar to that reported for N-BAR domain-containing proteins [13].

Using the three preparation methods described above, we have explored the effects of liposomes with broad size distributions and distinct lamellar properties on the membrane sculpting abilities of F-BAR domain proteins. Even though RSE liposomes have much lower average lamellarity than FT liposomes, the membrane morphologies generated by F-BAR proteins were similar in both liposome preparations under our experimental conditions. To further investigate how membrane curvature may govern membrane deformation activities of pacsin-1, pacsin-1^F-BAR^ and pacsin-1-PRD complex, we prepared liposomes of different size distributions by extruding FT or RSE liposomes through polycarbonate membranes with defined pore sizes. Extrusion produces unilamellar liposomes with well-defined sizes if the pore size used is ≤100 nm; with pore sizes ≥200 nm, the resulting liposomes contain mixed lamellarity and broader size distribution [65]. Extrusion is also another popular method for preparing liposomes, and hence provides a valuable comparison to the approaches described above.

Starting with FT or RSE liposomes, we used five membrane pore sizes for extrusion: 1000 nm, 800 nm, 400 nm, 200 nm, and 100 nm. DLS revealed narrow size distributions for 100 nm and 200 nm extruded liposomes, but broader distributions as the filter pore size increased (Fig. S8A–B). The size distributions and mean hydrodynamic sizes were comparable in extrusion preparations from FT or RSE liposomes, with narrower distributions at 100 nm and 200 nm, and slightly lower mean values when extruding from RSE liposomes (Fig. S8B). The mean liposome diameters obtained from 100 nm and 200 nm filter pores were ∼120 nm and ∼160 nm respectively, but increased only gradually when larger pore sizes were employed (Fig. S8C). This is consistent with the trends reported previously [66]. For the largest pore size (1000 nm), the mean liposome size obtained was only ∼360 nm, indicating the lack of direct correlation between extrusion filter pore size and actual liposome sizes obtained, when the filter pores ≥200 nm were used (Fig. S8C).

**Figure 8:**
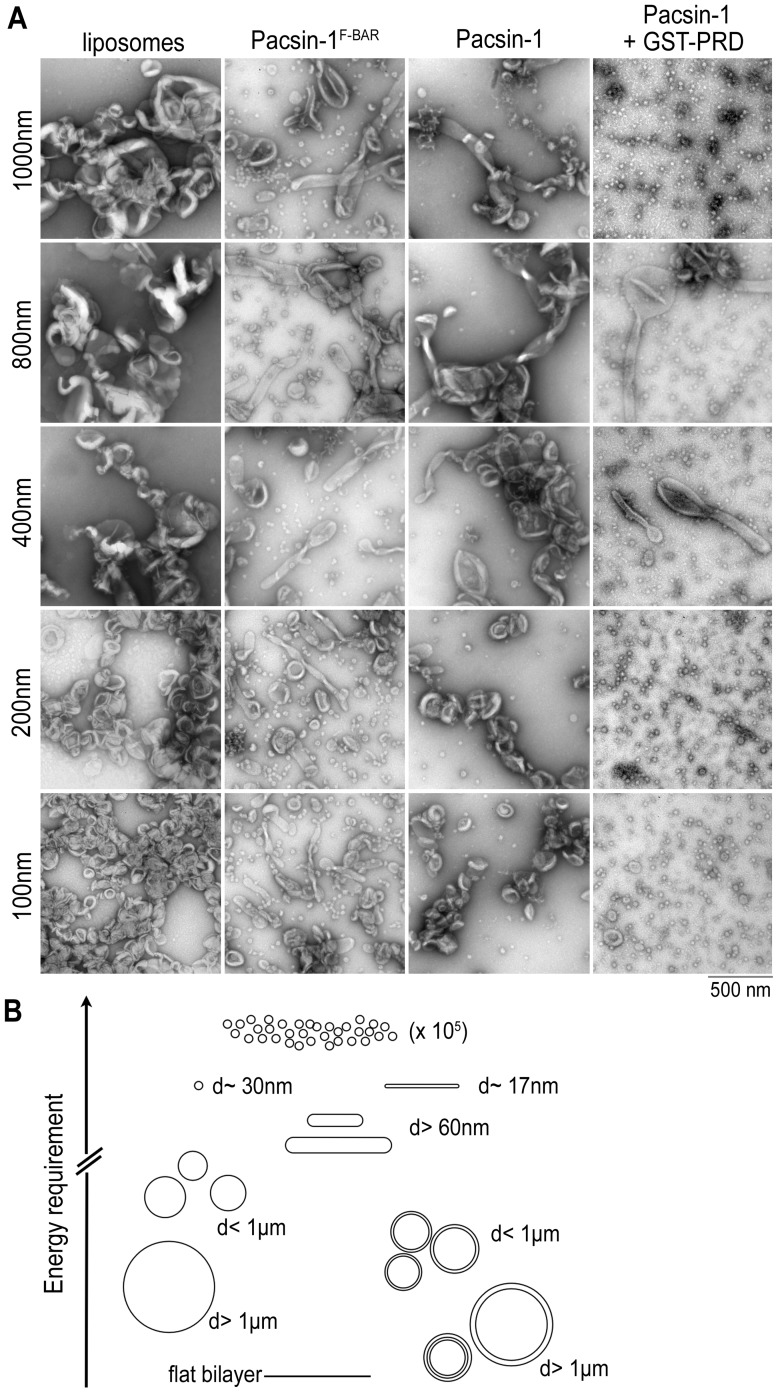
Effect of liposome diameter on protein-induced membrane deformation. A. Negative-stain EM of extruded liposomes. Folch liposomes were prepared using the freeze-thaw (FT) method, followed by extrusion using pore sizes ranging from 100–1000 nm. Protein incubations and imaging was carried out as described above. B. Model of modulated, protein-induced membrane deformation potential. The schematic diagram illustrates the energies required to generate various membrane morphologies, which is likely dependent on the system's initial energy state. Considering only membrane properties and a constant number of lipid molecules in each system, more energy is needed to generate a defined number of smaller vesicles from larger, multi-lamellar liposomes, compared to smaller, uni-lamellar liposomes as the starting material. The system may also be subject to bimodality, where distinct structures (vesicle vs. tubule) could coexist as energetically equivalent structures.

We examined the *in vitro* membrane deformation activity of pacsin-1, pacsin-1^F-BAR^ and pacsin-1 in the presence of GST-PRD in all extruded liposome preparations. In general, the membrane sculpting abilities of the proteins were not markedly different in liposomes that were extruded from FT or RSE liposomes. Pacsin-1^F-BAR^ generated wide tubules and short pearlings in all sizes of extruded liposomes (Fig. 8, Fig. S9). On the other hand, pacsin-1 displayed low tubulation activity in liposomes that were extruded through 1000 nm, 800 nm and 400 nm filter pores (Fig. 8, Fig. S9). Almost no tubules were observed in the 200 nm and 100 nm liposome preparations, indicating a strong dependence of pacsin-1's tubulation activity on membrane curvature. The membrane deformation activity of pacsin-1 in the presence of GST-PRD was comparable in all sizes of extruded liposomes, whereby tubular structures were produced in a background of small vesicles. The morphology of the vesicles generated were similar to those produced from FT and RSE liposomes (Fig. 7b), and the overall activity was not strongly dependent on the curvature of starting liposomes.

These comparative studies demonstrate that properties of the starting materials can influence the membrane remodeling potential of BAR domain proteins *in vitro*. While there is no ultimate superior method of preparing liposomes for *in vitro* membrane deformation assays, the observation that different preparation methods can give rise to various liposome properties can aid in the investigation of protein-induced membrane deformations. Multi-lamellarity not only reduces the effective liposome surface area that is exposed for protein binding, but also results in stiffer membranes (higher membrane bending modulus) that require more energy for remodeling. In addition, certain BAR domain-containing proteins are also sensitive to size (curvature) of the liposomes [21], [27]; for example, full-length pacsin-1 is more active with larger liposomes as starting material. Under our experimental conditions, pacsin-1 displayed increased ability to generate more vesicular structures in the presence of GST-PRD, even when given a wide range of liposome sizes. We also observed that the canonical F-BAR protein CIP4^F-BAR^ preferentially tubulates large liposomes, consistent with previous studies [21]. Contrastingly, pacsin-1^F-BAR^ appeared to have a broader curvature preference, generating variable membrane structures depending on the type of liposomes available. This versatility could be important in determining the role of pacsin-1 in membrane trafficking at the synapse.

### Energetic considerations for pacsin-mediated membrane tubulation and vesiculation

N-BAR-mediated tubulation is predominantly driven by the insertion of amphipathic helices into the head group-acyl chain interface of one membrane leaflet [17]–[19]. Based on theoretical estimations, protein shape and protein-membrane electrostatic interactions provide only a minor component to curvature generation [33], [67]. Consistently, similar calculations applied to pacsin-1^F-BAR^ showed that while charge and shape complementarity between pacsin-1 and the membrane may contribute sufficient energy to stabilize wide and even narrow tubules, they may not account for the generation of vesicular structures [27].

Experimentally, the membrane deformation potential of pacsin-1^F-BAR^ is sensitive to mutations in the amphipathic wedge loop and ionic strength of the buffer, indicating a mechanism involving the insertion of its wedge loop into one leaflet of the bilayer [27]. In order to assess the contribution of hydrophobic insertions on pacsin-mediated membrane sculpting, we estimated the wedge loop bending potential, taking into account the coupling of the two membrane leaflets (details are described in Text S1) [33]. The quantity ε describes the ratio between the surface of the outer and inner leaflet upon membrane bending. Insertion of motifs such as amphipathic helices or loops will counteract the initial surface mismatch. A comparison of ε and the surface area of these inserted motifs to the total membrane interaction surface of the protein scaffold provide an estimate of the energetic contribution from the wedge loop insertion to the bending process.

The N-BAR domains of endophilin and amphiphysin can effectively convert flat lipid bilayers into tubules with a radius R∼20–25 nm *in vitro*. Based on the crystal structures of the two proteins, their insertion motifs of the N-BAR domains account for approximately 50% and 25% (0.5 and 0.25, expressed as fractions) of the total membrane interaction interface, respectively [13], [17], [20], [33]. Both values are higher than the estimated excess surface ratio (ε∼0.17–0.22) that would initially be generated by the bending of a flat membrane to a tube with a radius, R = 20–25 nm (Text S1). This implies that the hydrophobic units in N-BAR domains are sufficient to counteract the surface area mismatch in a bent bilayer, which is consistent with the conclusions that have been drawn from the elastic model of laterally coupled monolayers [33].

Unlike the amphipathic helices in amphiphysin that span a total area of 12 nm^2^, the two wedge loops in the dimeric F-BAR domain of pacsin span only an area of 1.5 nm^2^. Assuming the extreme case of 100% membrane coverage with the F-BAR domain, the highest surface occupancy of these insertion units is only 5%–7% of the total membrane interaction interface. This number is far below the required ε∼0.31–0.5 for a tube with radius, R = 10–15 nm (Text S1). Similar conclusions can be made based on the elastic model of membrane monolayers. On an uncoupled monolayer, 10–12% of the membrane surface must be occupied by the insertion motif in order to generate a curvature with R = 10 nm. Based on these estimations, it is unlikely that pacsin's potential of generating highly curved membrane morphologies is solely driven by the insertion mechanism.

## Discussion

Bending membranes requires energy because bilayers tend to resist shape changes. Proteins contribute to this energy requirement via electrostatic interactions, scaffolding mechanisms, and the insertion of amphipathic helices or hydrophobic loops. Based on energetic considerations described above, it appears likely that the F-BAR domain of pacsin-1 relies on both scaffolding and hydrophobic insertions to deform membranes, with neither mechanism alone having the capacity to effectively shape membranes into the structures that were observed in our *in vitro* experiments. Both mechanisms may contribute additively to the deformation of membranes by counteracting the area mismatch between the two leaflets that would arise upon curvature generation, and by stabilizing a preferred membrane topology that is compatible with the shape imposed by the protein structure [27]. A mismatch in geometry between proteins and the membrane would destabilize their interaction, leading to potential disruption of protein lattices and changes in membrane remodeling propensities.

During membrane remodeling, the system's total free energy is a summation of the protein-membrane interaction energy and the internal energies of the protein and bilayer:
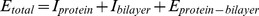



We assume that within the scope of elastic Gaussian theory, both internal energy terms are constant. Consequently, the membrane-protein interaction represents the major variable energy term in the system. From plotting the radii of tubes and vesicles against the energy density (energy per area) required for protein-induced membrane deformations, it is obvious that there are energetically equivalent structures that could potentially coexist (Text S1; illustrated in Fig. 8B). According to this simple approximation, a cylindrical structure (tubule) with a radius R and a spherical structure (vesicle) with a radius 2R represent systems with theoretically identical surface free energy density (Text S1). These estimations suggest that the system can be subject to bimodality, producing either narrow membrane tubules or vesicular structures, consistent with our experimental observations. Bimodality, in contrast to bistability, does not assume an identical origin. Indeed, we observed markedly different membrane morphologies induced by full-length pacsin-1, pacsin-1^F-BAR^ or PRD-bound pacsin-1 depending on the method that was used to prepare the initial liposomes. Differences in the frequency by which tubules and vesicles occur may be caused by differences of the initial liposome properties from which these structures arise.

Varying properties of the starting materials may elicit different apparent energetic barriers in the BAR protein-induced membrane deformation process. Variation in liposome properties such as lamellarity and size (curvature) could present different initial energy states that may dictate the likelihood of proteins to generate particular membrane morphologies in the system (Fig. 8B). For example, more energy is required to generate the same final number of 30 nm-vesicles from a system that initially contains larger liposomes than one that contains smaller liposomes (Text S1). Consistently, we observed that when given larger liposomes as the starting material (i.e. in FT and RSE liposomes, Fig. 7B), pacsin-1-PRD produced higher numbers of tubules and fewer vesicular structures, the latter being the dominant morphology when smaller liposomes (SFT liposomes) were provided as starting material.

Even though properties of the liposomes used in the *in vitro* assays could determine the outcome of the membrane sculpting process, the intrinsic structural characteristics of BAR domain proteins appear to be a main determinant of the range of morphologies that are generated. Under our experimental conditions, bimodality was observed in pacsin-1 and pacsin-1^F-BAR^ induced membrane morphologies, but not in the typical F-BAR protein, CIP4^F-BAR^. The versatile ability of pacsin-1^F-BAR^ to stabilize tubules of different sizes and invaginations is attributed to its S-shaped conformation (encoding two principal curvatures) and wedge loop insertion [27]. In contrast, CIP4^F-BAR^ only produced wide tubules regardless of the liposome properties, coinciding with a single principle curvature of the domain that prefers membranes of more shallow curvature [21], [23].

The autoinhibition of full-length pacsin-1 and the role of its SH3 domains in regulating its membrane deformation activity have been demonstrated both *in vivo* [38] and *in vitro* [27], [28]. Our liposome binding data showed that full-length pacsin-1 still interacts with membranes, albeit with decreased affinity, and prefers less-curved membranes compared to the isolated F-BAR domain. This may imply that other mechanisms, such as prevention of wedge loop insertion and/or protein oligomerization impair pacsin-1's membrane sculpting potential. In contrast, studies on endophilin-1 and its isolated N-BAR domain produced different results from comparable experiments with pacsin-1. Despite differences in membrane affinity, both full-length endophilin-1 and endophilin-1^N-BAR^ still retained potent tubulation activities. The presence of four amphipathic helices presented by the N-BAR domain suggests that the insertion mechanism dominates in endophilin-dependent membrane tubulation [17], [19]. Differences in the average tubule diameters produced by full-length endophilin-1 and endophilin-1^N-BAR^ could be due to different lateral protein-protein interactions, resulting in protein scaffolds with differing membrane curvature preferences.

While it is difficult to separate and quantify the relative contributions that affect protein-mediated membrane deformation, we demonstrate that high-affinity binding of the entire PRD to the SH3 domains of pacsin-1 results in a more efficient recruitment of the complex to membranes compared to that observed for the individual proteins. The high SH3-PRD affinity is dependent on intact core binding motifs in the PRD (aa768–792) [46], [52], as demonstrated by our pull-down and mutagenesis studies, and is required for the increased membrane deformation activities observed in pacsin-1 and endophilin-1. In short, binding of the PRD sequesters the SH3 domains away from the F-BAR or BAR domains, and appears to be the main mechanism of activation.

It is not immediately obvious why sequestration of SH3 domains away from the F-BAR or BAR domains would alter the membrane deformation capacity of the proteins. Yet, we identified several experimental conditions under which we observed tubulation with the isolated F-BAR and N-BAR domains, but vesiculation in the context of complexes containing full-length pacsin or endophilin and the PRD of dynamin-1. Binding of pacsin-1 to full-length GST-PRD (or SUMO-PRD) may have induced some formation of higher-order oligomers in the absence of membranes. We observed a small shoulder to the left of the pacsin-PRD main elution peak in the gel filtration profile (Fig. S3A), and also small amounts of the pacsin-PRD complex co-pelleting in the absence of liposomes (Fig. S5B). Pacsin-1's altered membrane sculpting propensity could be a result of the PRD-SH3 interactions further enabling the arrangement of pacsin-1 into higher-order oligomers on the membrane to facilitate deformation. The formation of oligomers on membranes has been reported in several independent studies conducted on other BAR domain proteins [23], [34], [68].

Another possible explanation for the increased membrane deformation activity of the complex is more efficient targeting of pacsin-1 and endophilin-1 to membranes, as facilitated by their interaction with dynamin's PRD, imposes local steric confinement that can drive membrane deformation. Indeed, steric confinement of membrane-bound green fluorescent proteins and the ENTH domain of epsin have been shown to induce tubulation from targeted domains on giant unilamellar vesicles [69], [70], a process that is also likely dependent on membrane properties [69], [71]. Electrostatic binding of positively charged surfaces from both pacsin-1 and PRD may locally attract acidic lipids, altering local membrane mechanical properties that may favor deformation. Furthermore, this enhanced complex-membrane interaction could stress the bilayer to an extent that it becomes unstable. This instability could lead to or promote vesiculation under additional perturbations, like those found in the negative staining procedure. In contrast, tubular structures such as those stabilized by endophilin's N-BAR domain, have a high degree of curvature yet remain stable under the experimental conditions used here, suggesting a PRD-complex specific phenomenon. Recently, Boucrot *et. al.* [72] showed that shallow insertions of amphipathic helices by epsin's ENTH domains are sufficient for vesiculation, whereas the presence of BAR domain scaffolds could limit the full potential of hydrophobic insertions, such as in endophilin and amphiphysin. It is possible that the limiting effect of the BAR scaffold could be diminished upon complex formation between the full-length proteins and the PRD, thus unleashing the vesiculation potential of the proteins driven by shallow hydrophobic insertions under certain conditions.

Even under the simple, minimalist conditions of *in vitro* experiments, we have identified multiple factors that could affect the thermodynamics and kinetics of membrane remodeling. In cells, the plasma membrane is constantly changing due to various cellular processes. As an adaptable and efficient membrane sculptor, regulated by its SH3 module, pacsin-1 could function at various stages during membrane trafficking. The importance of pacsin-1 is evident in synaptic vesicle recycling, where it recruits dynamin to fission sites [73]. Furthermore, multiple studies have revealed a principle role for pacsin-1 at the synapse during high neuronal activity [40], [42], [74]. In that scenario, dephosphorylation of dynamin-1 on its PRD leads to complex formation with pacsin-1 and an increase in bulk endocytosis [40]. One hypothesis is that pacsin may contribute in a more regulated and direct fashion to the endocytotic capacity of a cell under certain conditions, likely facilitated by the actin cytoskeleton, a model that will need further corroboration. In addition, cellular membranes may differentially attract a distinct subset of BAR domain-containing proteins in a curvature-dependent manner. Alternatively, a difference in local curvatures may determine the outcome of BAR domain-mediated membrane interactions.

Our *in vitro* experiments provide simple models that suggest a synergistic relationship between pacsin (or endophilin) and dynamin in membrane remodeling, which is an energetically expensive process that is dependent on both protein structural characteristics and membrane properties. The detailed molecular mechanism of their interplay in facilitating membrane fission is only beginning to be unraveled.

## Materials and Methods

### Lipids

1-palmitoyl, 2-oleoylphosphatidylethanolamine (POPE), 1-palmitoyl, 2-oleoylphosphatidylcholine (POPC) and 1-palmitoyl, 2-oleoylphosphatidylserine (POPS) were purchased from Avanti Polar Lipids. Folch fraction I lipids were purchased from Sigma.

### Protein expression and purification

Human, full-length pacsin isoforms 1–3, pacsin-1^F-BAR^ (residues 1–325), pacsin-2^F-BAR^ (residues 1–324), pacsin-3^F-BAR^ (residues 1–322), pacsin-1^P437L^ and mouse full-length endophilin-A1 and endophilin^N-BAR^ (residues 1–256) were produced following standard molecular biology and liquid chromatography techniques. The coding regions of the pacsin and endophilin constructs described above were amplified by standard PCR and cloned into a modified pET28a expression plasmid (Novagen) yielding N-terminally hexahistidine-tagged SUMO fusion proteins. The hexahistidine-tagged SUMO-moiety was cleavable by addition of the protease Ulp-1 from *S. cerevisiae*. Proteins were expressed and purified as described previously [27], [35].

Mouse dynamin-1 PRD (residues 747–842) in the expression vector pGEX-6P-1 was kindly provided by the De Camilli laboratory (Yale University). Truncation mutants (GST-PRD^trunc1^ and GST-PRD^trunc2^) were generated by amplifying the corresponding regions by standard PCR. Point mutants were produced using site-directed mutagenesis (Stratagene Quikchange). All PRD constructs were expressed and purified as GST fusion proteins (resin: GSTrap, GE Healthcare), following the manufacturer's instructions. GST fusion proteins were subjected to size exclusion chromatography on a Superdex 200 column (GE Healthcare) equilibrated in gel filtration buffer (25 mM Tris- HCl, pH 7.5, 150 mM NaCl). Proteins were concentrated in a Centricon ultrafiltration device (10 kDa cutoff; Millipore) to a final concentration of ∼0.5–1 mM (∼25–50 mg/ml). Protein aliquots were frozen in liquid nitrogen and stored at −80°C.

### Liposome preparation via the freeze/thaw method (FT)

Folch fraction I lipids (Sigma) were dissolved in chloroform and stored at −20°C. Appropriate amounts of lipids were dispensed into glass tubes, followed by evaporation of chloroform under a stream of nitrogen gas until a film was formed. Samples were subjected to high vacuum to remove residual organic solvent (final gauge reading ∼35 mTorr). The dry lipid film was resuspended in buffer (25 mM Tris-HCl, pH 7.5, 50 mM NaCl) to a concentration of 2−10 mg/ml by alternate vortexing and short incubations in a 45°C water bath. Finally, the hydrated liposomes were subjected to five freeze-thaw cycles using liquid nitrogen.

### Liposome preparation via the sonication/freeze-thaw method (SFT)

Hydrated liposomes resuspended from dried film (as described above) were sonicated to clarity in a bath sonicator (Laboratory Supplies Co., Inc), followed by 8–10 freeze-thaw cycles using liquid nitrogen. Liposomes were incubated at 30°C for 1 hour before use in negative stain EM or liposome co-pelleting experiments.

### Liposome preparation via the rapid solvent exchange method (RSE)

Liposomes were prepared according to procedures described in Buboltz and Feigenson [64], and modified as previously described [75]. Briefly, lipids in chloroform solution were dispensed into glass tubes. After the addition of buffer (25 mM Tris-HCl, pH 7.5, 50 mM NaCl), the mixture was vortexed under vacuum for one minute and then sealed under argon gas, yielding 2–10 mg/mL hydrated liposomes for negative-stain EM or liposome co-pelleting experiments.

### Liposome extrusion

Hydrated liposomes prepared from either the FT or RSE method were extruded 21–41 times through polycarbonate filters of pore sizes ranging from 100 nm to 1000 nm (Avestin, Inc). Extruded liposomes were used on the day of extrusion.

### Dynamic Light Scattering

Size distributions of various liposome preparations (0.5 mg/ml) were measured using dynamic light scattering on a Malvern Zetasizer Nano-ZS. A minimum of three measurements were made per sample. The mean liposome size for each extrusion preparation was calculated from the frequency distribution curve as:
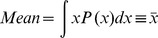



The skewness of the each distribution was analyzed as:
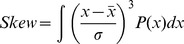
where σ is the standard deviation of the distribution. The skew value becomes increasingly positive for filter pore sizes ≥400 nm, indicating a broader size distribution containing larger liposomes. Based on the average skewness calculated for each extrusion preparation, representative distribution curves are shown (Fig. S8).

### Liposome co-pelleting assay

Equal volumes of liposomes (0.5 mg/ml) and proteins (5–10 μM) were incubated in 40 μl low salt buffer for 20 min at room temperature. Samples were centrifuged in an Optima MAX-E ultracentrifuge (Beckman) equipped with a TLA-100 rotor at 87,000 rpm at 4°C for 1 hour. After carefully removing supernatant, pellets were resuspended in 40 μl low salt buffer, and both fractions were analyzed by SDS-PAGE. Gels were stained with SYPRO Ruby (Sigma) and quantified using Image J.

### Liposome flotation assay

100 nm-extruded liposomes (8 mg/ml) and proteins (8–15 μM) were incubated in 25 μl low salt buffer (25 mM Tris-Cl, pH7.5, 50 mM NaCl) for 20 min at room temperature. 75 μl of 70% (w/v) sucrose that was prepared in the same buffer was added to each reaction. After mixing, 80 μl of the mixture was placed at the bottom of a 250 μl ultracentrifuge tube. This was then overlaid with 90 μl of 40% (w/v) sucrose, and 40 μl of 4% (w/v) sucrose. Samples were centrifuged in an Optima MAX-E ultracentrifuge (Beckman) equipped with a TLA-100 rotor at 87,000 rpm at 4°C for 1 hour. After centrifugation, 7×30 μl fractions were then carefully removed from the top to the bottom of each tube and analyzed by SDS-PAGE. Gels were stained with SYPRO Ruby (Sigma) and quantified in Image J. Proteins or protein complexes found in the top three fractions were indicated as membrane bound.

### Negative Staining Electron Microscopy (EM)

Liposomes (1 mg/ml) made from Folch fraction I (bovine) brain lipids (Sigma) or synthetic lipids (27.5/27.5/45  =  POPC/POPE/POPS, Avanti Polar Lipids, Inc.) were incubated in the presence or absence of proteins (5–10 μM, unless indicated otherwise) in low salt buffer for 5 min at room temperature. The sample was applied to a carbon-formvar-coated copper grid (Electron Microscopy Sciences) and incubated for two minutes. Excess liquid was carefully removed by blotting with a wet Kimwipe (Kimberly-Clark). The grids were stained three times with 6 μl of 2% filtered uranyl acetate solution, blotted immediately after each stain application. Samples were air-dried before imaging. Membrane morphologies were examined on a FEI Morgagni Transmission Electron Microscope with the electron energy set to 80 kV. Representative images were taken on an AMT camera with a direct magnification of 18kx–44kx. Liposome size measurements and quantitation of vesicle size distributions were performed using ImageJ.

### GST pull-down experiments

50 μl pre-packed GST resin (GST SpinTrap, GE Healthcare) was washed with binding buffer (25 mM Tris-Cl, pH 7.5, 100 mM NaCl). Various purified GST-PRD recombinant proteins were incubated with the resin for 1 hour at 4°C with gentle rocking. Excess unbound proteins were removed by centrifuging for 30 s at 100 *g*. The resin was then washed 5 times with binding buffer. Various purified pacsin-1 “prey” proteins were then incubated with the resin for 2 h at 4°C with gentle rocking. Unbound proteins were removed via centrifugation and the resin was washed 5 times. Finally, all proteins were eluted from the resin with elution buffer (10 mM glutathione, 25 mM Tris-Cl, pH 7.5, 100 mM NaCl) after 10 min incubation.

## Supporting Information

Figure S1 **Vesiculation activity of pacsin-1 in the presence of GST-PRD is found over a wide range of protein concentrations.** Negative-stain electron micrographs were taken on Folch liposomes incubated with increasing concentrations of GST-PRD (constant full-length pacsin-1), A, of full-length pacsin-1 (constant GST-PRD), B, or of pacsin-1/GST-PRD complexes, C.
(TIF)

Figure S2 **Activation of pacsin-1 occurs in the presence of SUMO-PRD.** A. Negative-stain EM images of Folch liposomes following incubation with the indicated proteins or protein complexes as described previously. B. Representative images of SDS-PAGE gels from liposome co-pelleting assays. Proteins or protein complexes were co-incubated with Folch liposomes, and the amounts of proteins in the supernatant and pellet (membrane bound) fractions were analyzed, as described in Materials and Methods.
(TIF)

Figure S3 **Co-migration of pacsin-1 and GST-PRD in size exclusion chromatography (SEC) indicates formation of a stable complex.** A. Wild-type pacsin-1. Human wild-type pacsin-1 (40 µM) and GST-PRD (80 µM) were incubated for 15 min, and subjected to size-exclusion chromatography. Protein-containing fractions were analyzed by using SDS-PAGE and Coomassie staining. B. Pacsin-1^P437L^. A similar analysis was carried out with a single-point mutant of pacsin-1, in which the peptide binding site is disrupted.
(TIF)

Figure S4 **Mutation of the wedge loop affects the vesiculation of pacsin-1 in the presence of GST-PRD.** Negative-stain EM images of Folch liposomes incubated with the wedge loop mutant, pacsin-1^M126K^ alone or in the presence of GST-PRD.
(TIF)

Figure S5 **Representative images of SDS-PAGE gels from liposome co-pelleting assays.** The amounts of proteins in the supernatant and pellet fractions were analyzed in the presence of Folch liposomes (0.5 mg/ml), A, and in the absence of liposomes, B. Gels were stained with SYPRO Ruby and experiments were conducted as described in Materials and Methods.
(TIF)

Figure S6 **Analysis of the membrane binding affinity of pacsin-1 constructs and pacsin-PRD complexes.** Liposome flotation assays were employed to assess the amount of membrane bound proteins. The horizontal, dashed lines indicate the lipid-bound fraction of the isolated pacsin-1 F-BAR domain under similar conditions. A. Membrane bound fractions of wild-type pacsin-1 and GST-PRD in isolation and in complex. B. Membrane bound fractions of pacsin-1^P437L^ and GST-PRD in isolation and in complex. C. Similar experiments and analysis as in (A) and (B), examining the membrane bound fractions of pacsin-1 in the presence of GST-PRD mutants. The horizontal solid line indicates the lipid-bound fraction of the isolated full-length pacsin-1 under similar conditions as shown in (A). Error bars represent standard deviations of a minimum of 3 independent experiments.
(TIF)

Figure S7 **Size distribution of vesicles generated by pacsin-1 in the presence of wild-type and various GST-PRD mutants.** A. Vesicles produced by pacsin-1 with wild-type or GST-PRD^trunc1^. The mean diameters are not significantly different based on a two-tailed unpaired t-test (p<0.1, 750<N<1400). B. Vesicles produced by pacsin-1 in the presence of GST-PRD^ArgKO1^ and GST-PRD^ArgKO2^. Vesicle diameters produced by the mutant GST-PRD variants are significantly different from the wild-type case based on a two-tailed unpaired t-test (p<0.0001 for GST-PRD^ArgKO1^; p<0.005 for GST-PRD^ArgKO2^; 750<N<1300).
(TIF)

Figure S8 **Dynamic light scattering analysis on the size distribution of liposomes produced by extrusion.** A. Representative frequency distributions of FT liposomes that were extruded through various filter pore sizes. Narrower distributions were observed with 100 nm and 200 nm pore size. B. Representative frequency distributions of RSE liposomes that were extruded through various filter pore sizes. Similar to (A), narrower distributions were found using 100 nm and 200 nm pore sizes compared to larger pore sizes. C. Mean liposome diameters calculated from the intensity distributions of extruded RSE and FT liposomes are similar for each pore size. Standard deviations are shown for N≥3 measurements at each filter pore size. With the exception of 100 nm and 200 nm pore sizes, the mean diameter obtained is always smaller than the actual pore size used. The mean for non-extruded RSE and FT liposomes was ∼450 nm.
(TIF)

Figure S9 **Effect of liposome diameter on protein-induced membrane deformation.** Negative-stain EM of extruded liposomes. Folch liposomes were prepared using the rapid solvent exchange (RSE) method, followed by extrusion using different pore sizes ranging from 100–1000 nm. Protein incubations and imaging was carried out as described before.
(TIF)

Text S1 (DOC)
