# Supplementary figures and images for: Versatile Membrane Deformation Potential of Activated Pacsin
Source: PLoS One. 2012 Dec 7;7(12):e51628. doi: 10.1371/journal.pone.0051628 (PMC3517540; doi:10.1371/journal.pone.0051628)

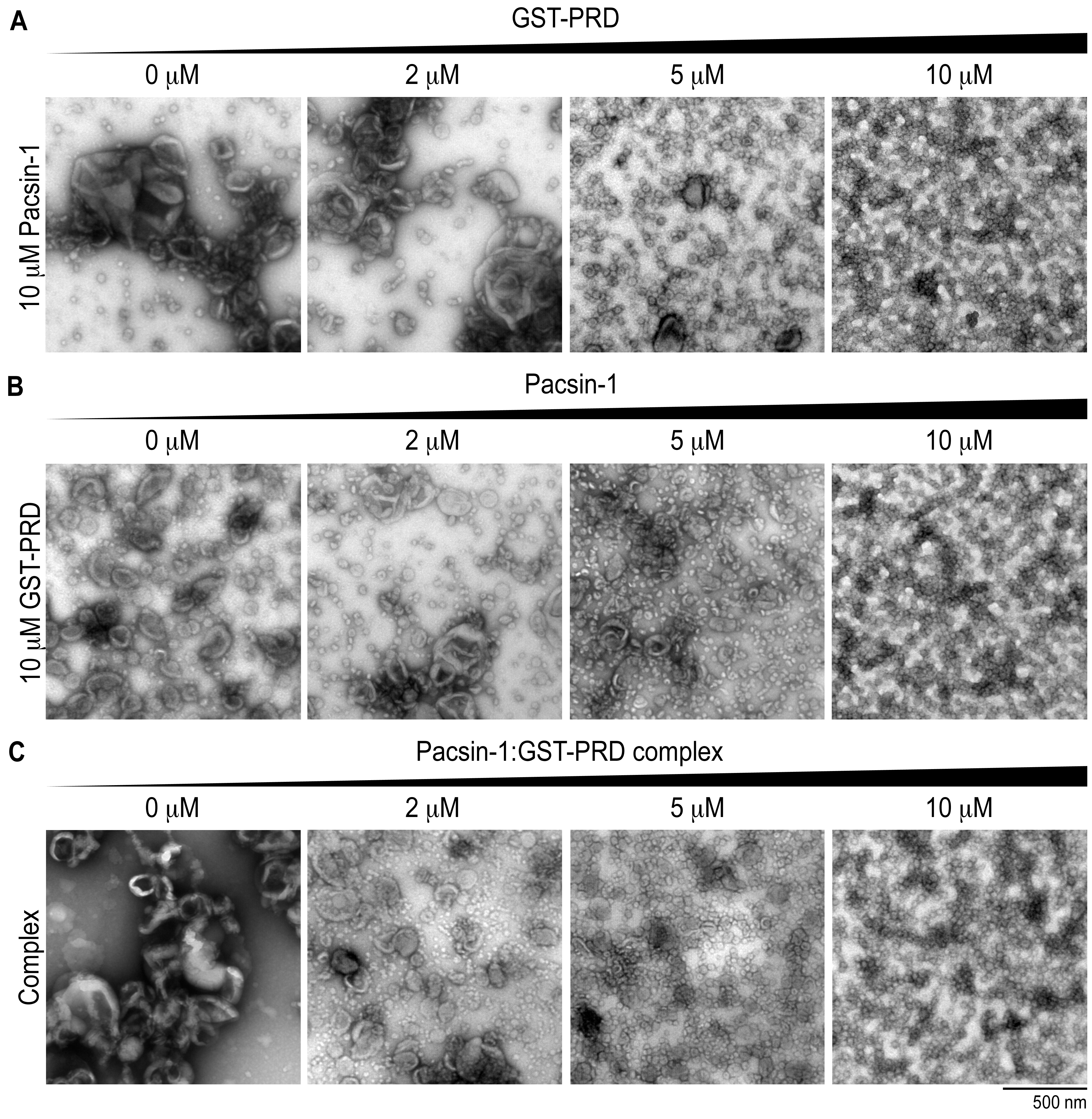

Supplement: Figure S1 — Vesiculation activity of pacsin-1 in the presence of GST-PRD is found over a wide range of protein concentrations. Negative-stain electron micrographs were taken on Folch liposomes incubated with increasing concentrations of GST-PRD (constant full-length pacsin-1), A, of full-length pacsin-1 (constant GST-PRD), B, or of pacsin-1/GST-PRD complexes, C. (TIF) [file pone.0051628.s001.tif]

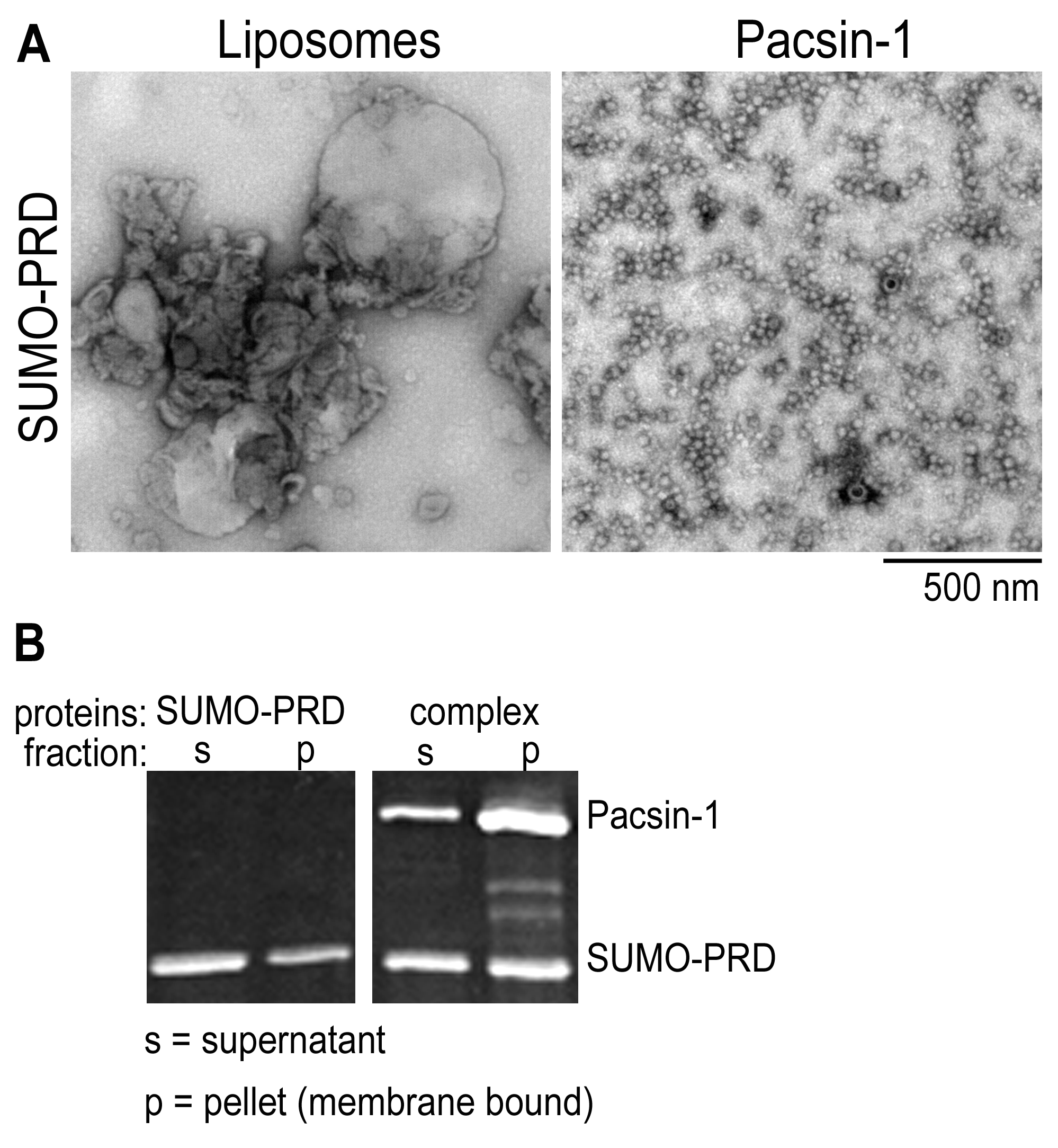

Supplement: Figure S2 — Activation of pacsin-1 occurs in the presence of SUMO-PRD. A. Negative-stain EM images of Folch liposomes following incubation with the indicated proteins or protein complexes as described previously. B. Representative images of SDS-PAGE gels from liposome co-pelleting assays. Proteins or protein complexes were co-incubated with Folch liposomes, and the amounts of proteins in the supernatant and pellet (membrane bound) fractions were analyzed, as described in Materials and Methods. (TIF) [file pone.0051628.s002.tif]

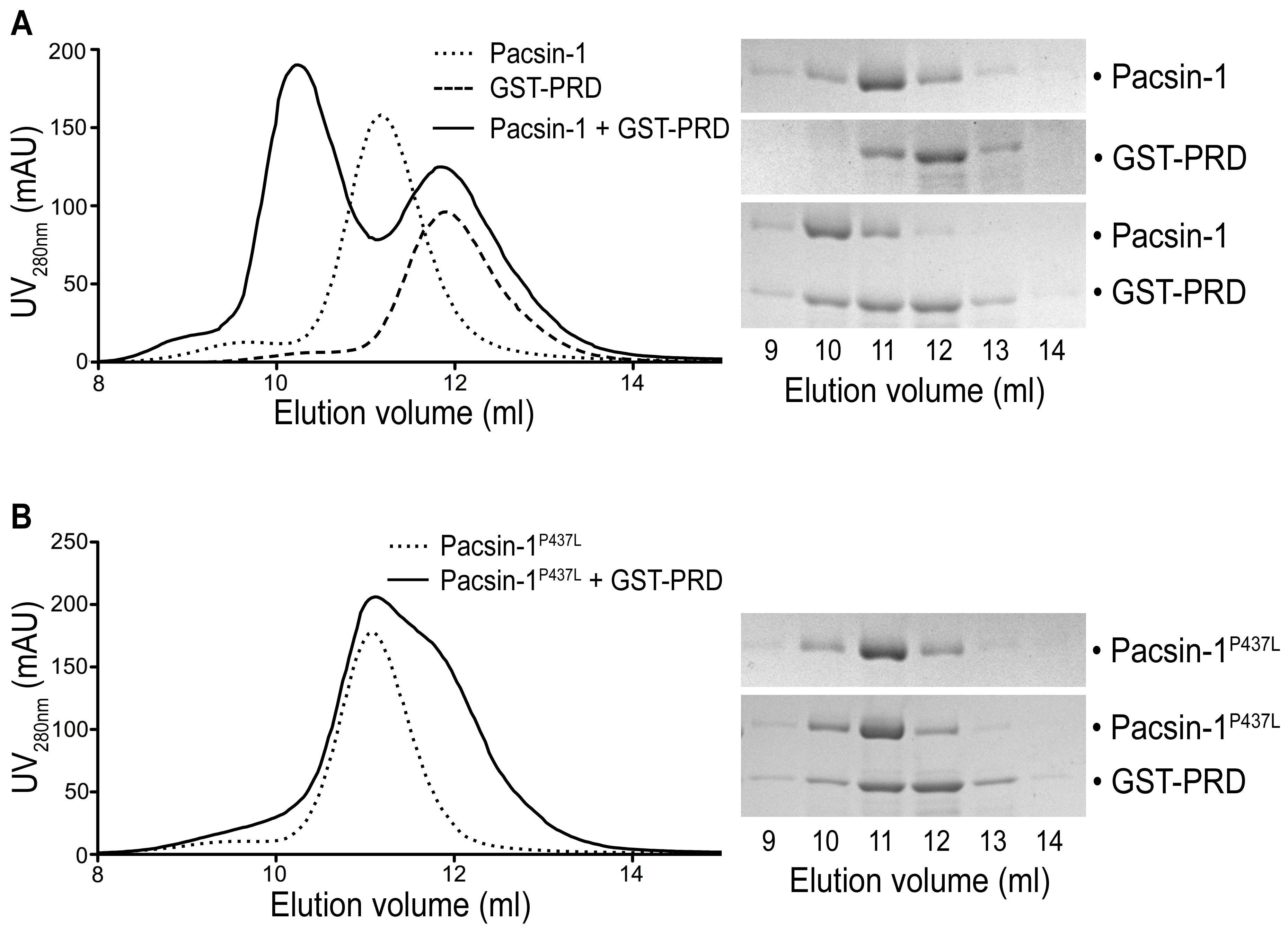

Supplement: Figure S3 — Co-migration of pacsin-1 and GST-PRD in size exclusion chromatography (SEC) indicates formation of a stable complex. A. Wild-type pacsin-1. Human wild-type pacsin-1 (40 µM) and GST-PRD (80 µM) were incubated for 15 min, and subjected to size-exclusion chromatography. Protein-containing fractions were analyzed by using SDS-PAGE and Coomassie staining. B. Pacsin-1P437L. A similar analysis was carried out with a single-point mutant of pacsin-1, in which the peptide binding site is disrupted. (TIF) [file pone.0051628.s003.tif]

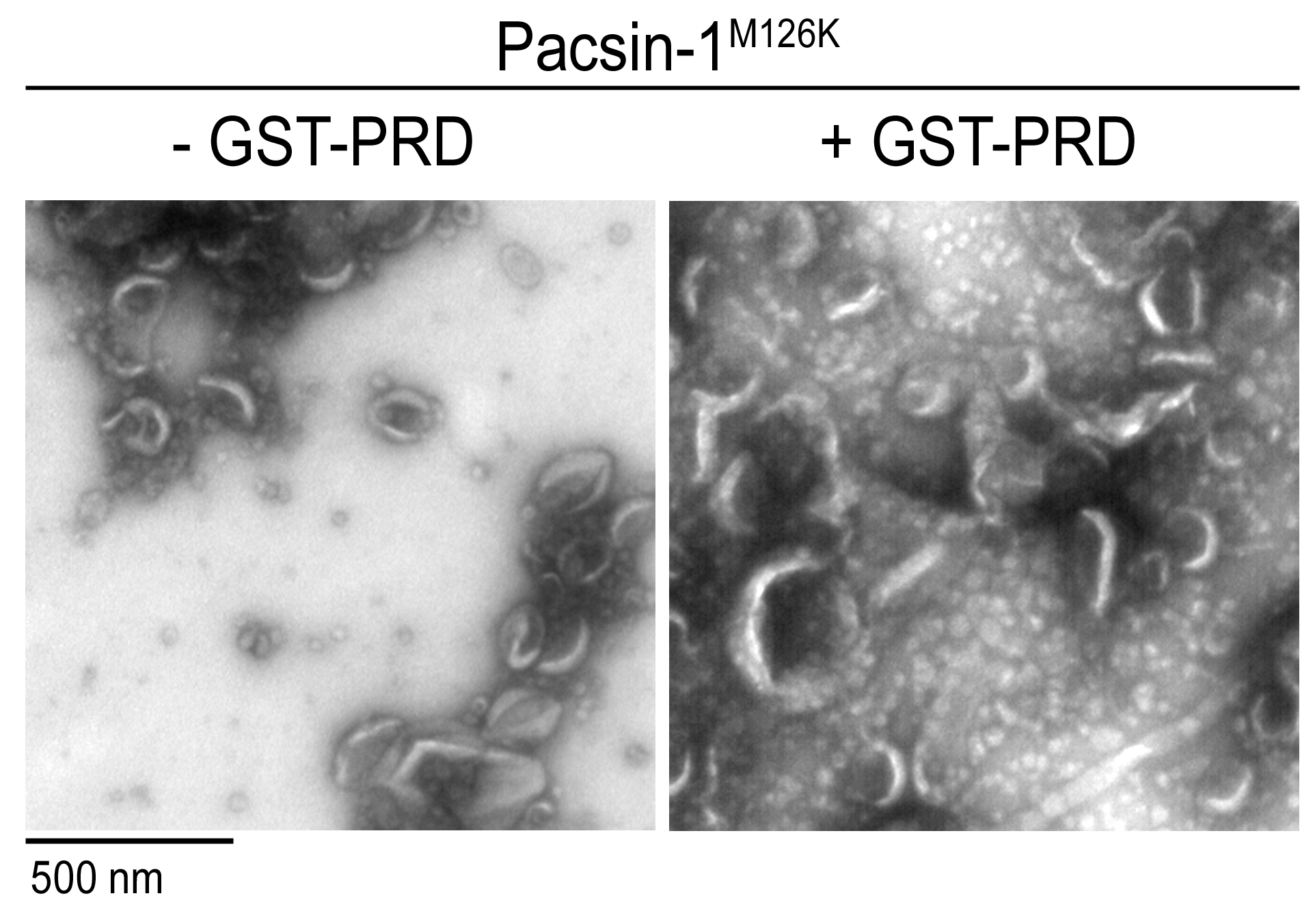

Supplement: Figure S4 — Mutation of the wedge loop affects the vesiculation of pacsin-1 in the presence of GST-PRD. Negative-stain EM images of Folch liposomes incubated with the wedge loop mutant, pacsin-1M126K alone or in the presence of GST-PRD. (TIF) [file pone.0051628.s004.tif]

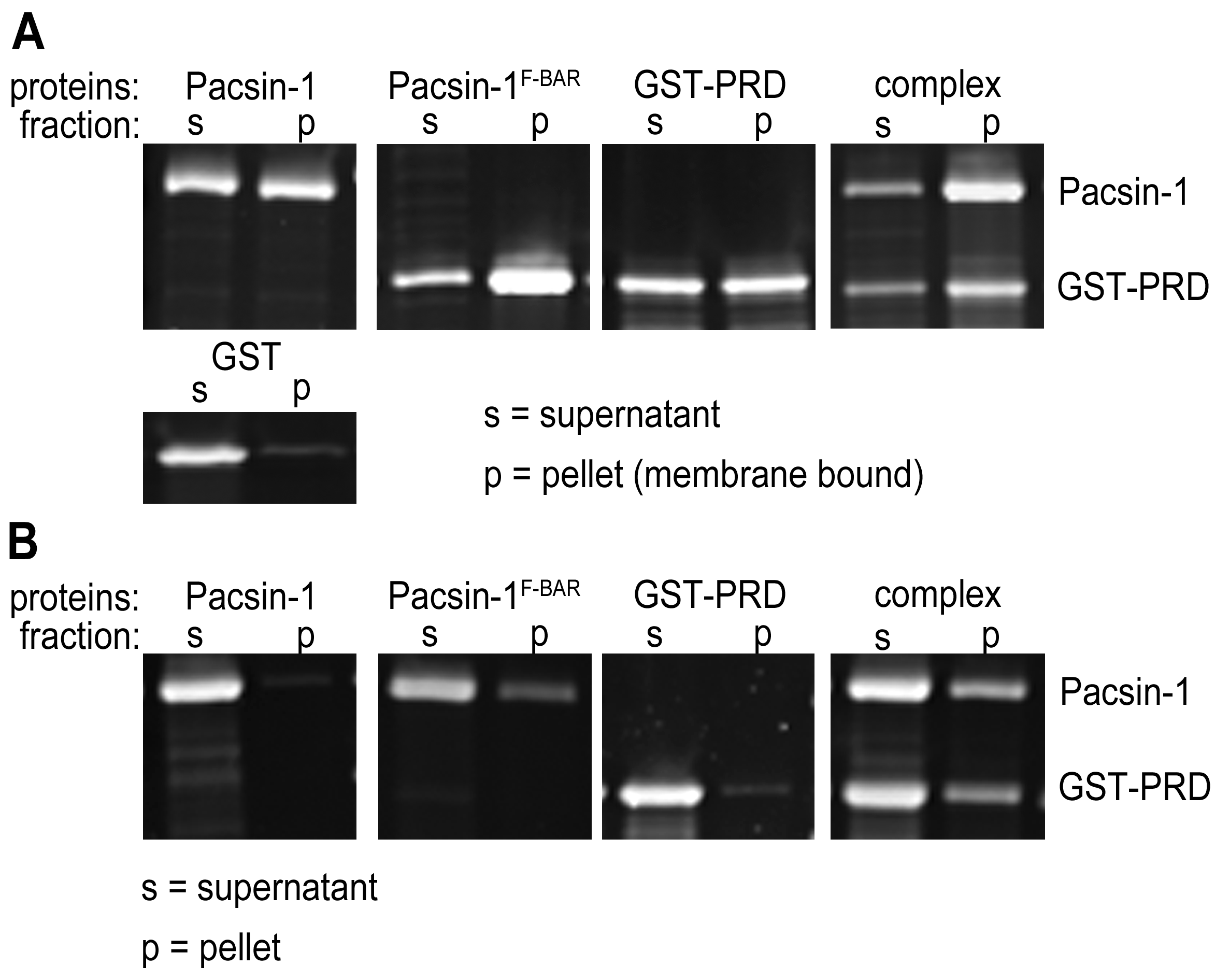

Supplement: Figure S5 — Representative images of SDS-PAGE gels from liposome co-pelleting assays. The amounts of proteins in the supernatant and pellet fractions were analyzed in the presence of Folch liposomes (0.5 mg/ml), A, and in the absence of liposomes, B. Gels were stained with SYPRO Ruby and experiments were conducted as described in Materials and Methods. (TIF) [file pone.0051628.s005.tif]

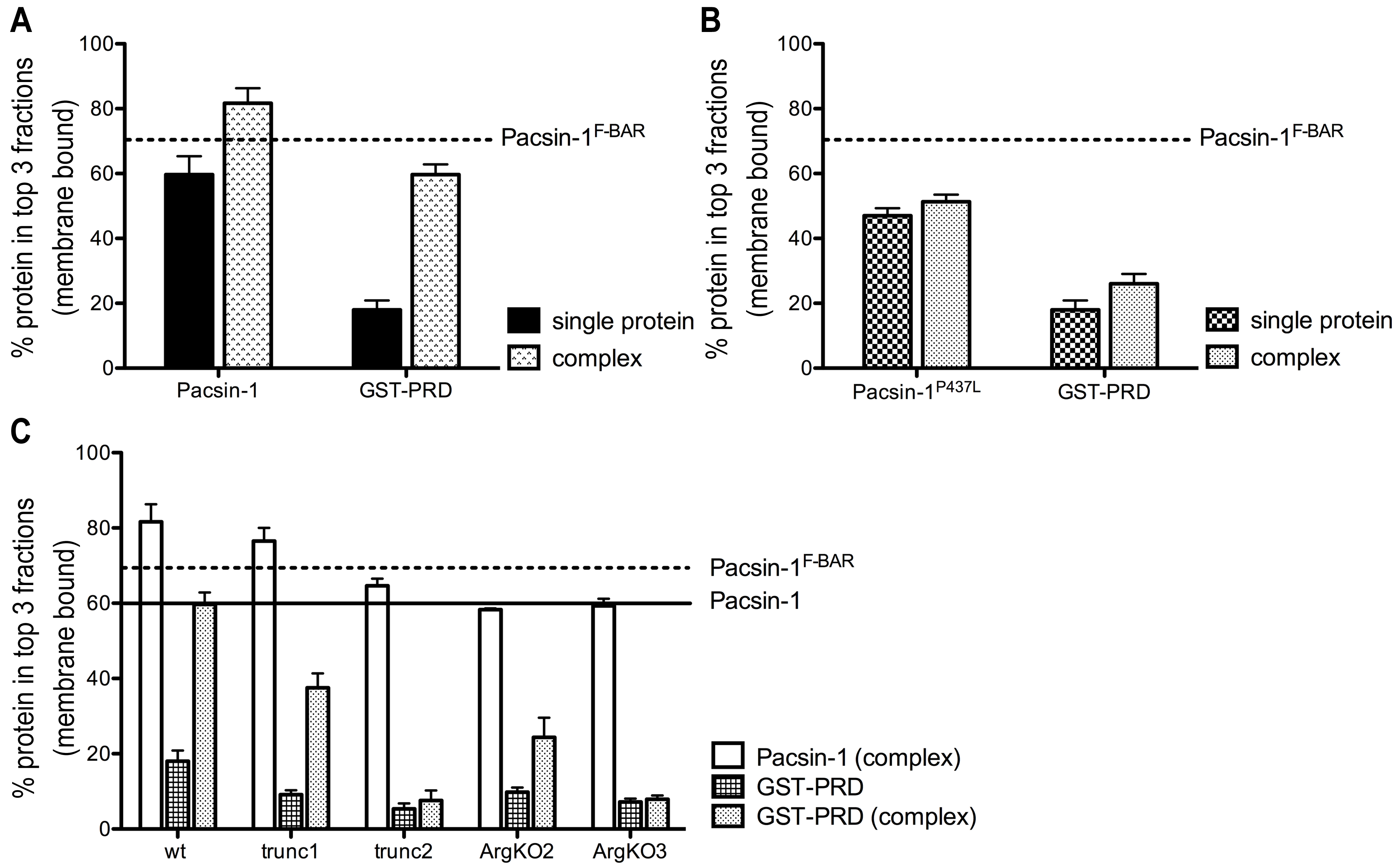

Supplement: Figure S6 — Analysis of the membrane binding affinity of pacsin-1 constructs and pacsin-PRD complexes. Liposome flotation assays were employed to assess the amount of membrane bound proteins. The horizontal, dashed lines indicate the lipid-bound fraction of the isolated pacsin-1 F-BAR domain under similar conditions. A. Membrane bound fractions of wild-type pacsin-1 and GST-PRD in isolation and in complex. B. Membrane bound fractions of pacsin-1P437L and GST-PRD in isolation and in complex. C. Similar experiments and analysis as in (A) and (B), examining the membrane bound fractions of pacsin-1 in the presence of GST-PRD mutants. The horizontal solid line indicates the lipid-bound fraction of the isolated full-length pacsin-1 under similar conditions as shown in (A). Error bars represent standard deviations of a minimum of 3 independent experiments. (TIF) [file pone.0051628.s006.tif]

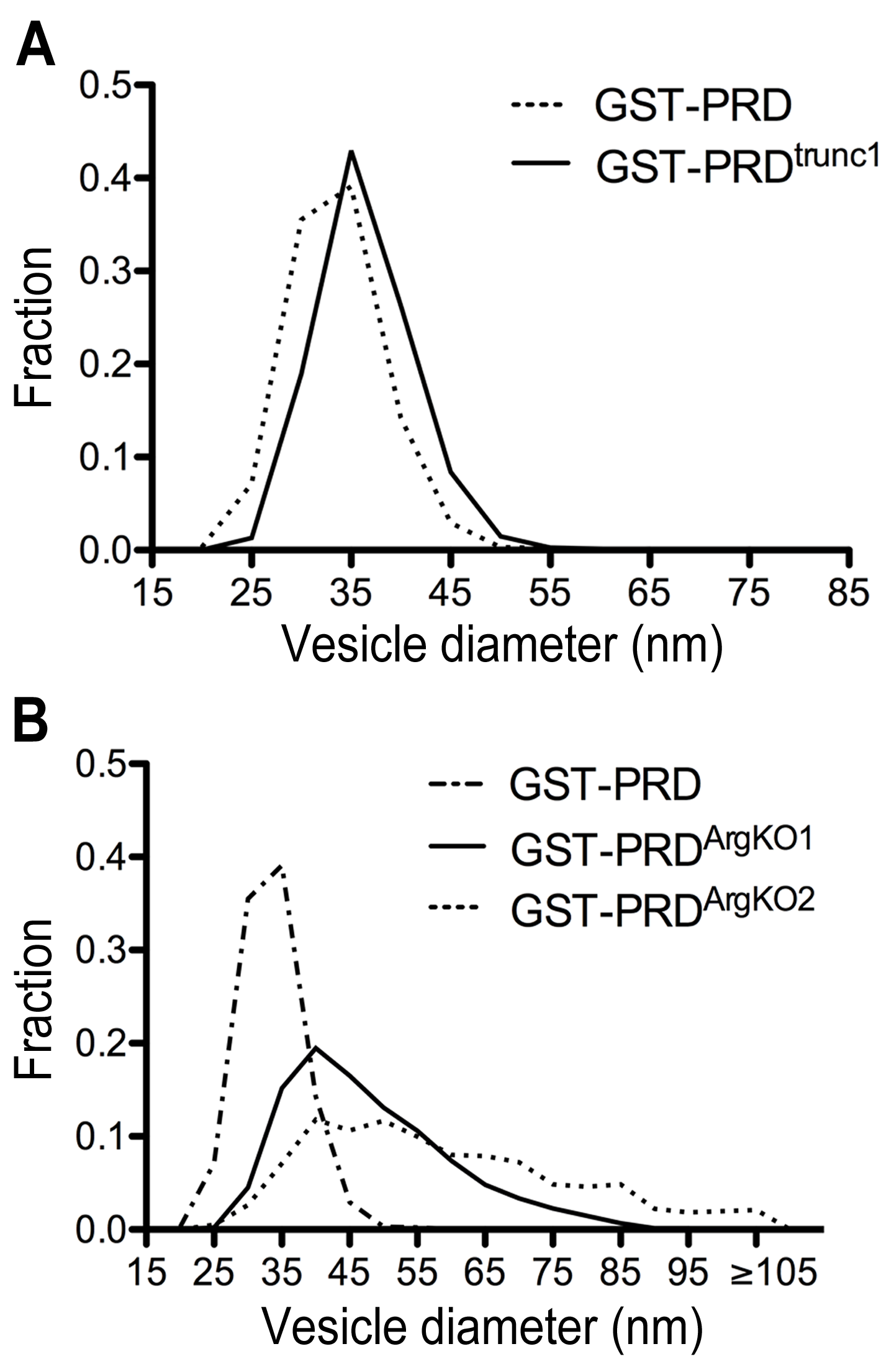

Supplement: Figure S7 — Size distribution of vesicles generated by pacsin-1 in the presence of wild-type and various GST-PRD mutants. A. Vesicles produced by pacsin-1 with wild-type or GST-PRDtrunc1. The mean diameters are not significantly different based on a two-tailed unpaired t-test (p<0.1, 750<N<1400). B. Vesicles produced by pacsin-1 in the presence of GST-PRDArgKO1 and GST-PRDArgKO2. Vesicle diameters produced by the mutant GST-PRD variants are significantly different from the wild-type case based on a two-tailed unpaired t-test (p<0.0001 for GST-PRDArgKO1; p<0.005 for GST-PRDArgKO2; 750<N<1300). (TIF) [file pone.0051628.s007.tif]

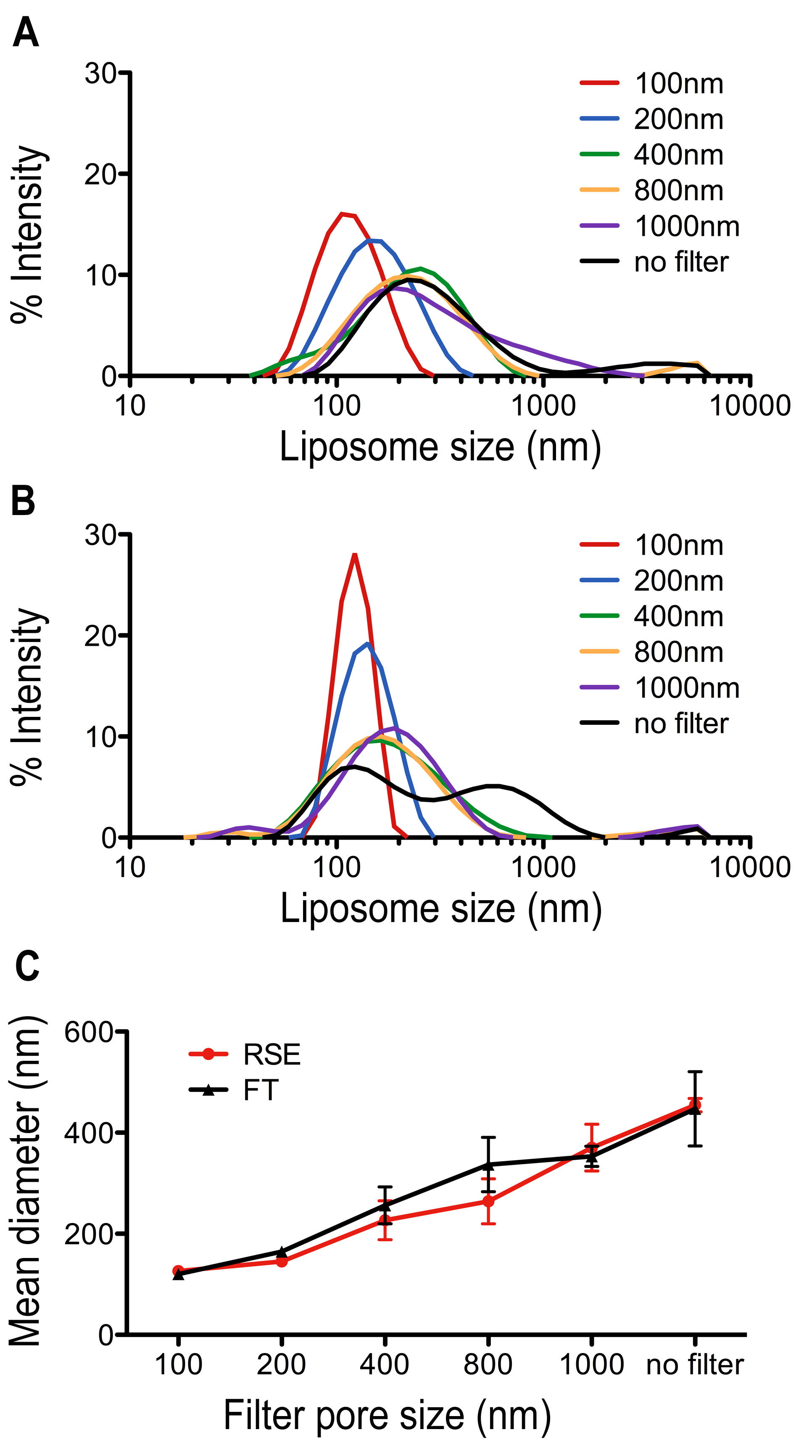

Supplement: Figure S8 — Dynamic light scattering analysis on the size distribution of liposomes produced by extrusion. A. Representative frequency distributions of FT liposomes that were extruded through various filter pore sizes. Narrower distributions were observed with 100 nm and 200 nm pore size. B. Representative frequency distributions of RSE liposomes that were extruded through various filter pore sizes. Similar to (A), narrower distributions were found using 100 nm and 200 nm pore sizes compared to larger pore sizes. C. Mean liposome diameters calculated from the intensity distributions of extruded RSE and FT liposomes are similar for each pore size. Standard deviations are shown for N≥3 measurements at each filter pore size. With the exception of 100 nm and 200 nm pore sizes, the mean diameter obtained is always smaller than the actual pore size used. The mean for non-extruded RSE and FT liposomes was ∼450 nm. (TIF) [file pone.0051628.s008.tif]

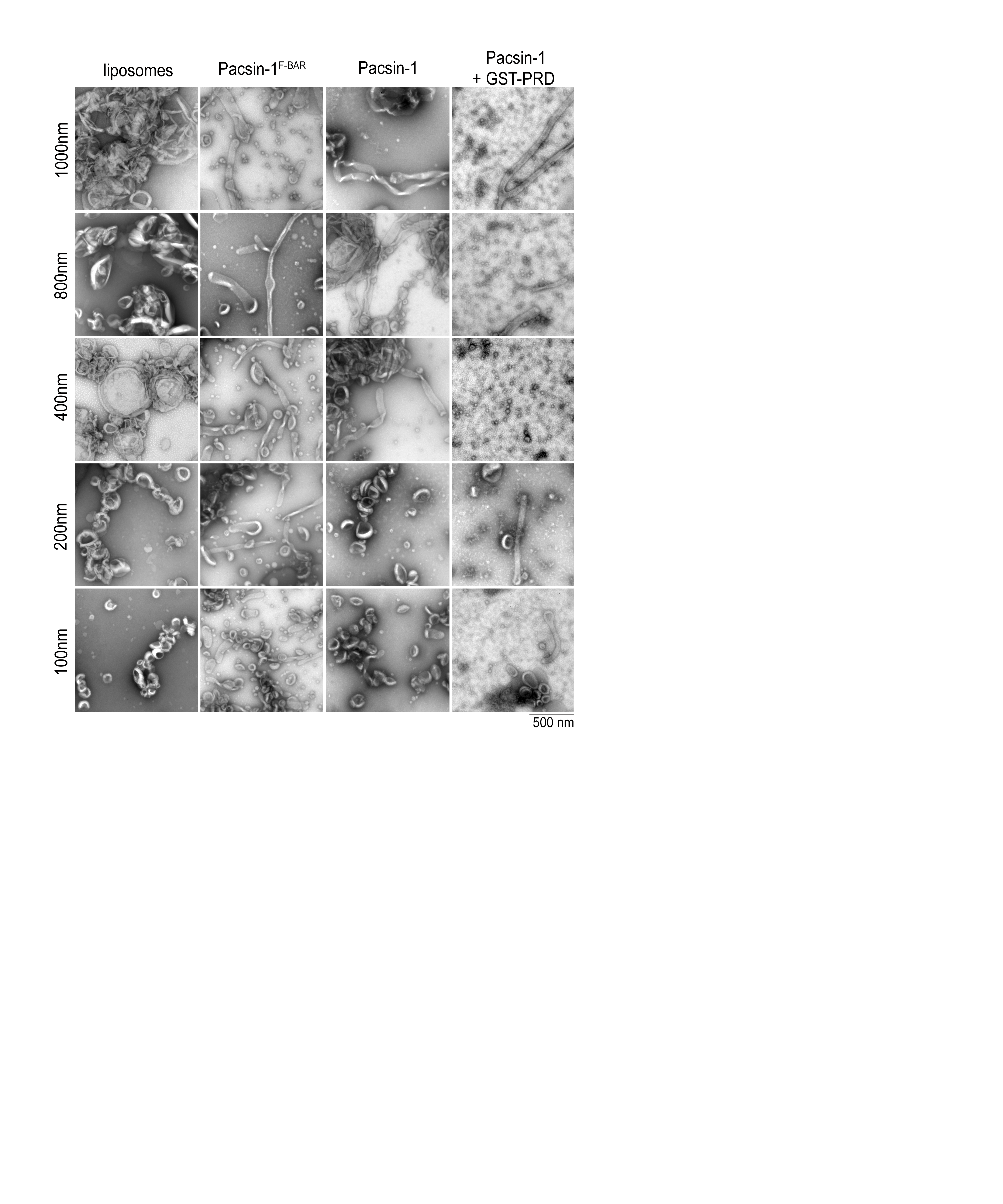

Supplement: Figure S9 — Effect of liposome diameter on protein-induced membrane deformation. Negative-stain EM of extruded liposomes. Folch liposomes were prepared using the rapid solvent exchange (RSE) method, followed by extrusion using different pore sizes ranging from 100–1000 nm. Protein incubations and imaging was carried out as described before. (TIF) [file pone.0051628.s009.tif]
