# Supplementary material for: Versatile Membrane Deformation Potential of Activated Pacsin
Source: PLoS One. 2012 Dec 7;7(12):e51628. doi: 10.1371/journal.pone.0051628 (PMC3517540; doi:10.1371/journal.pone.0051628)
Supplement: Text S1 — (DOC) [file pone.0051628.s010.doc]

**Supplemental Materials**

**Energy calculations and estimations for protein-mediated membrane deformation**

*Section 1.1*

The overall radius of a curved bilayer, considering the asymmetry of surface area of the two leaflets, is given by^1^:

$$R=d\times\frac{A^{out}+A^{in}}{A^{out}-A^{in}}$$

Reformulation of this equation gives the relationship between $A^{out}$ and $A^{in}$:

$$\frac{A^{out}}{A^{in}}=1+\frac{2d}{R-d}=1+\varepsilon$$

where $\varepsilon$ is the excess surface ratio of the two leaflets. This equation indicates that a more curved surface is accompanied by a higher degree of surface expansion at the outer-leaflet of the membrane. The table below lists the dependence of $\varepsilon$ on the membrane curvature.

| R (nm) | 5 | 10 | 15 | 20 | 25 | 30 | 35 | 40 | 45 | 50 | 55 | 60 |
| --- | --- | --- | --- | --- | --- | --- | --- | --- | --- | --- | --- | --- |
| $\varepsilon$ | 1.33 | 0.50 | 0.31 | 0.22 | 0.17 | 0.14 | 0.12 | 0.11 | 0.09 | 0.08 | 0.08 | 0.07 |

For a 10 nm tubular structure, the surface area of the outer-leaflet membrane is twice that of the inner-leaflet.

*Section 1.2*

The energy cost to generate a tube of diameter 2R_t_ and length L from a flat membrane can be described as:

$$E_{t}=\pi\kappa\frac{L}{R_{t}}$$

where κ is the membrane bending rigidity, generally estimated as 20 *k_B_T* for a plasma membrane. Therefore, the energy cost per unit area (energy density) will be:

$$\rho_{t}=\frac{E_{t}}{2\pi R_{t}L}=\frac{\kappa}{2}\times\frac{1}{R_{t}^{2}}$$

Similarly, the energy density to generate a spherical vesicle with a radius R_s_ nm from a flat membrane can be estimated as:

$$\rho_{v}=2\kappa\times\frac{1}{R_{v}^{2}}$$

At the isoenergy density point$\rho_{v}=\rho_{t}$ the following relation may apply:

$$R_{v}=2R_{t}$$

This relationship implies that a tubular structure of radius R has the same surface energy density as a vesicle with radius 2R, which forms the basis for our discussion on tubule-vesicle bimodality.

*Section 1.3*

The energy required to bend a flat symmetric bilayer into a sphere (*E_sph_*) is *8πκ*, independent of the size (radius) of the sphere^2^. Hence, the energy needed to make *N_f_* number of spheres is

*E = N_f_ x E_sph_*

If the initial state contains *N_i_* spheres, then the energy needed to make *N_f_* spheres is the difference between the energies of the two states:

*ΔE = N_f_ * E_sph_ - N_i_ * E_sph_ = (N_f_ - N_i_) E_sph_*

The process is more favorable if the difference between *N_i_* and *N_f_* is small. Hence, if two systems have the same total number of lipids, where system A is made up of larger liposomes (e.g. >1 micron), and system B consists of smaller liposomes (e.g. <1 micron), it will require more energy to generate the same final number of 30 nm diameter vesicles from system A than from system B.

**Supplementary Materials References**

1. Campelo, F., McMahon, H.T. & Kozlov, M.M. (2008). The Hydrophobic Insertion Mechanism of Membrane Curvature Generation by Proteins. *Biophys. J.* **95**, 2325–2339

2. Zimmerberg, J. & Kozlov, M.M. (2005). How proteins produce cellular membrane curvature. *Nat Rev Mol Cell Biol* **7**, 9–19
